# Supplementary figures and images for: A chemically induced attenuated strain of Candida albicans generates robust protective immune responses and prevents systemic candidiasis development
Source: eLife. 2024 May 24;13:RP93760. doi: 10.7554/eLife.93760 (PMC11126311; doi:10.7554/eLife.93760)

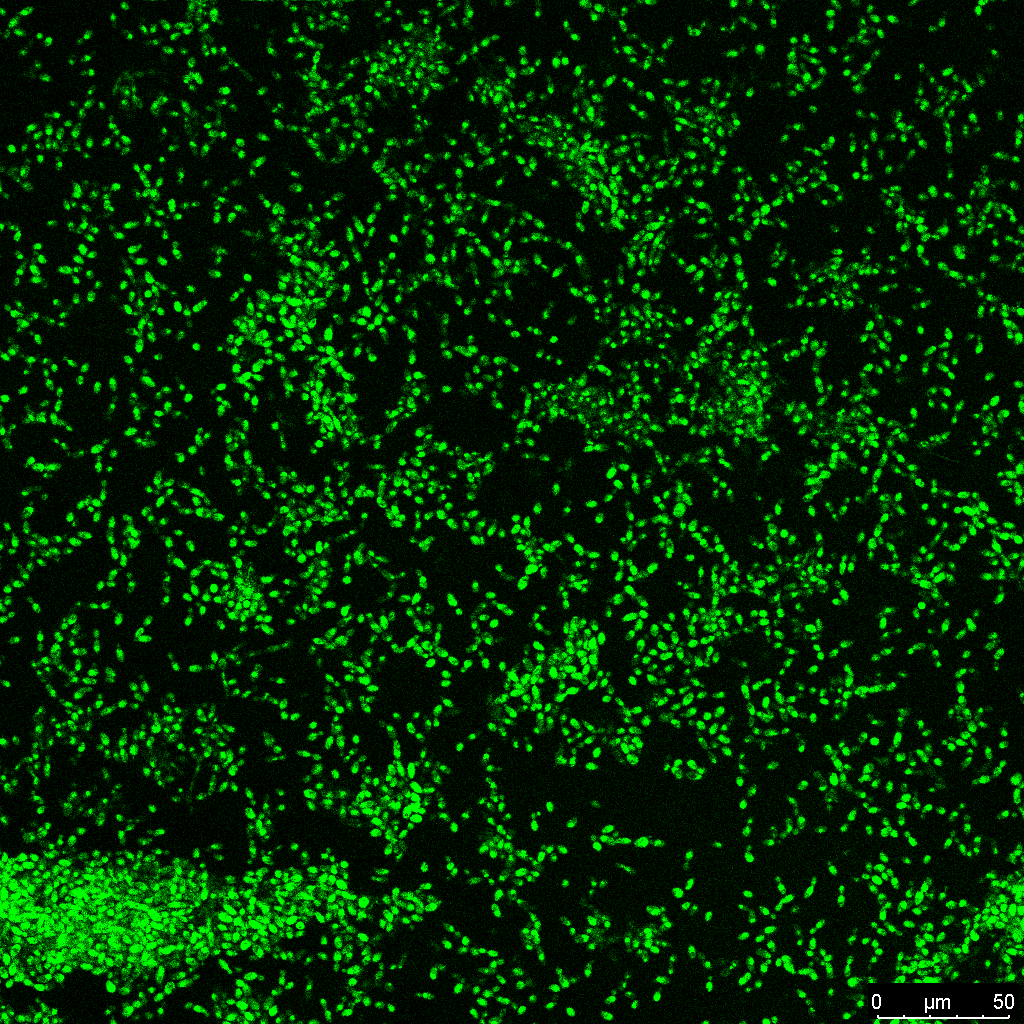

Supplement: Figure 1—source data 1. [file elife-93760-fig1-data1.zip › Figure 1/Figure D Biofilm Image/Fig1D Ca (2D picture).tif]

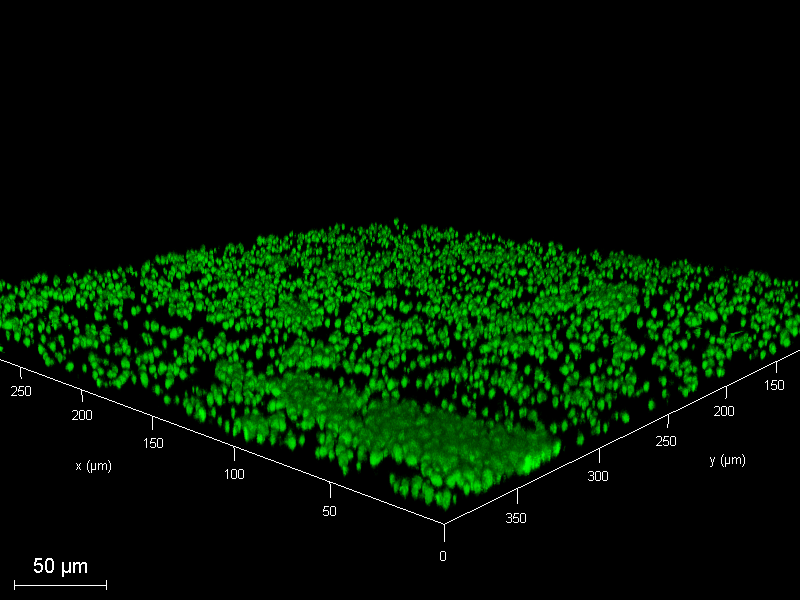

Supplement: Figure 1—source data 1. [file elife-93760-fig1-data1.zip › Figure 1/Figure D Biofilm Image/Fig1D Ca (3D picture).tif]

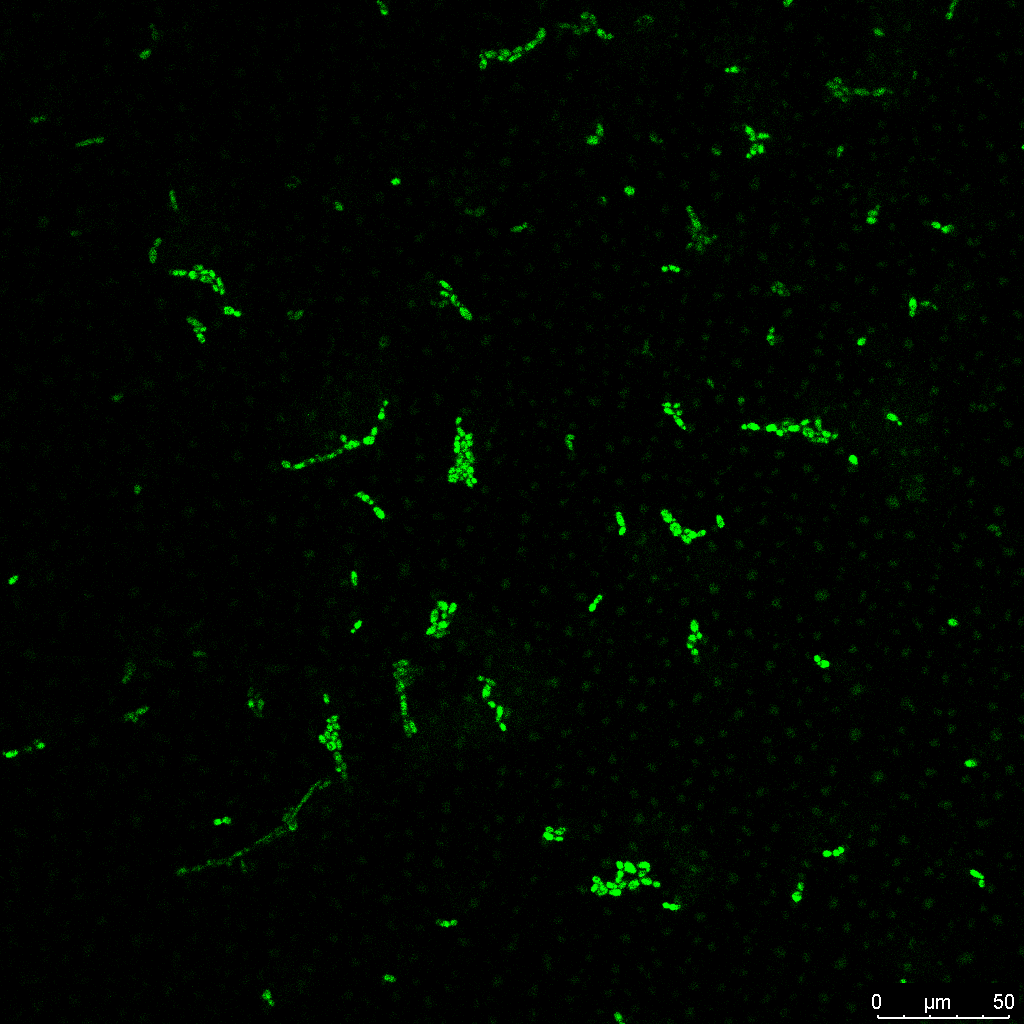

Supplement: Figure 1—source data 1. [file elife-93760-fig1-data1.zip › Figure 1/Figure D Biofilm Image/Fig1D Ca+250uMEDTA (2D picture).tif]

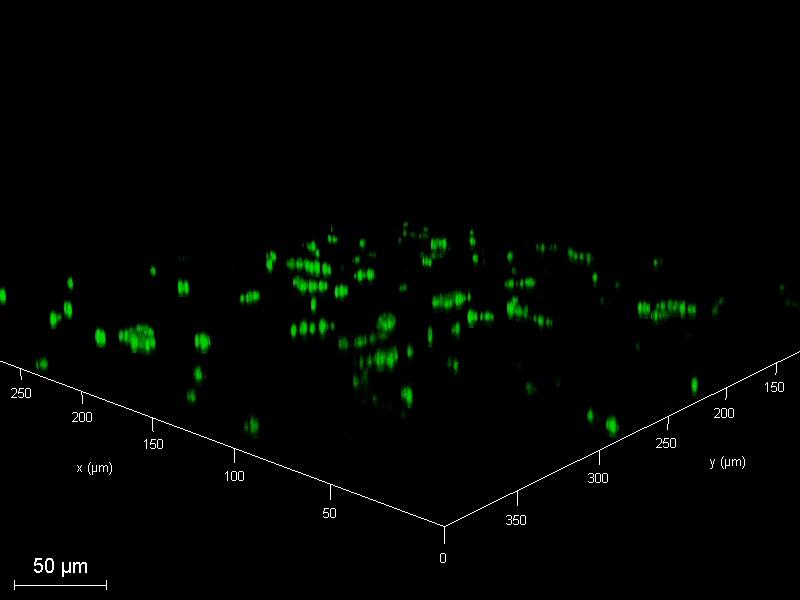

Supplement: Figure 1—source data 1. [file elife-93760-fig1-data1.zip › Figure 1/Figure D Biofilm Image/Fig1D Ca+250uMEDTA (3D picture).tif]

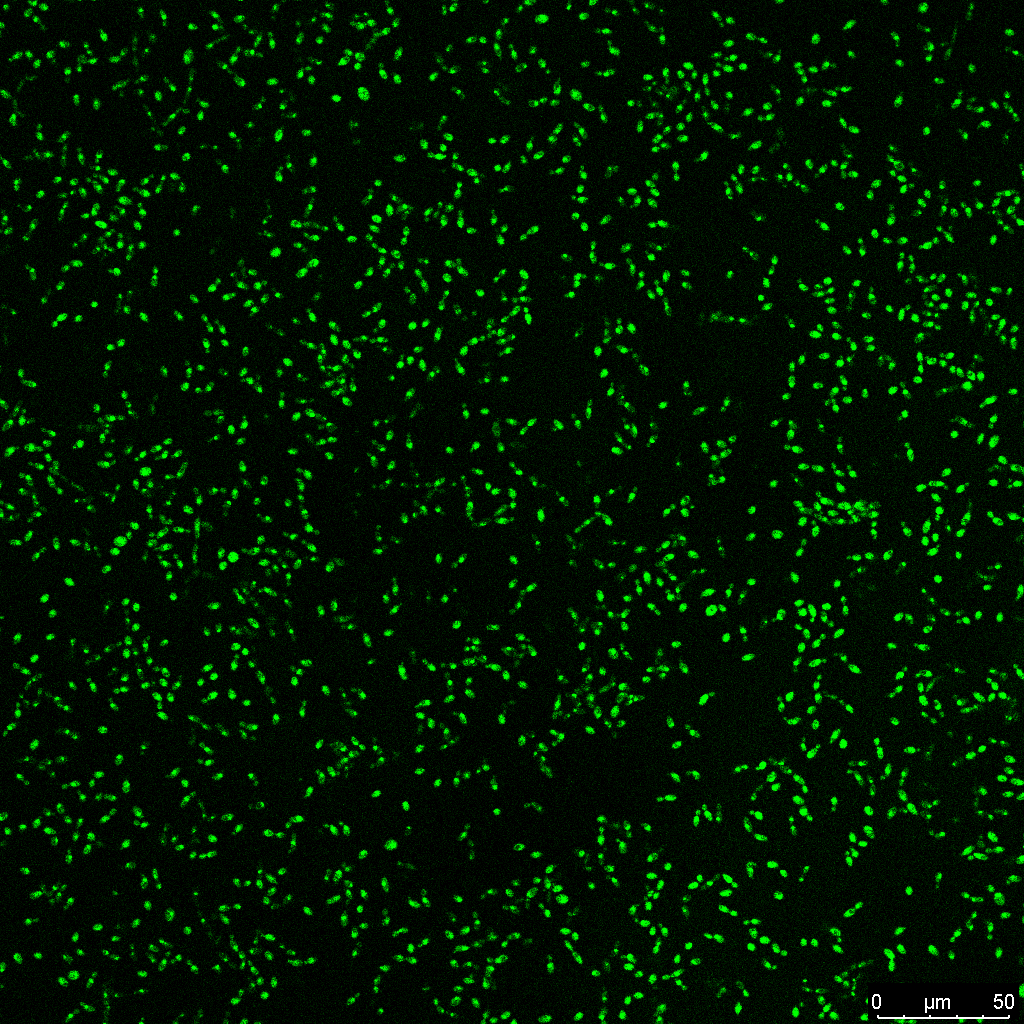

Supplement: Figure 1—source data 1. [file elife-93760-fig1-data1.zip › Figure 1/Figure D Biofilm Image/Fig1D Ca+250uMEDTA+8uMMgSO4 (2D picture).tif]

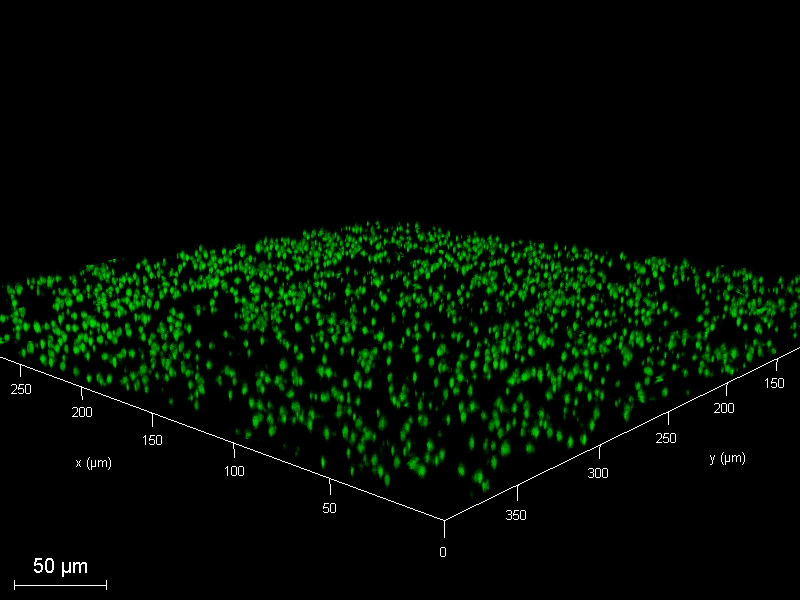

Supplement: Figure 1—source data 1. [file elife-93760-fig1-data1.zip › Figure 1/Figure D Biofilm Image/Fig1D Ca+250uMEDTA+8uMMgSO4 (3D picture).tif]

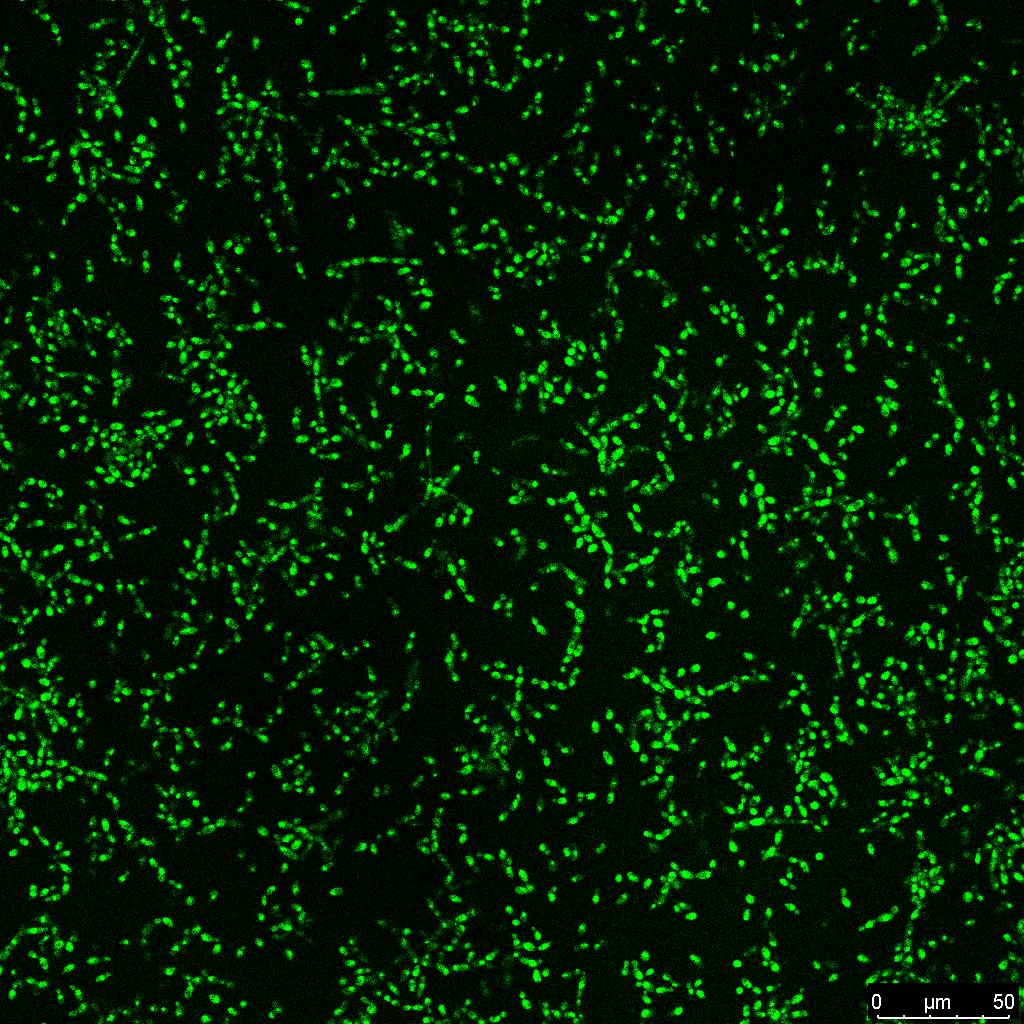

Supplement: Figure 1—source data 1. [file elife-93760-fig1-data1.zip › Figure 1/Figure D Biofilm Image/Fig1D Ca+8uMMgSO4 (2D picture).tif]

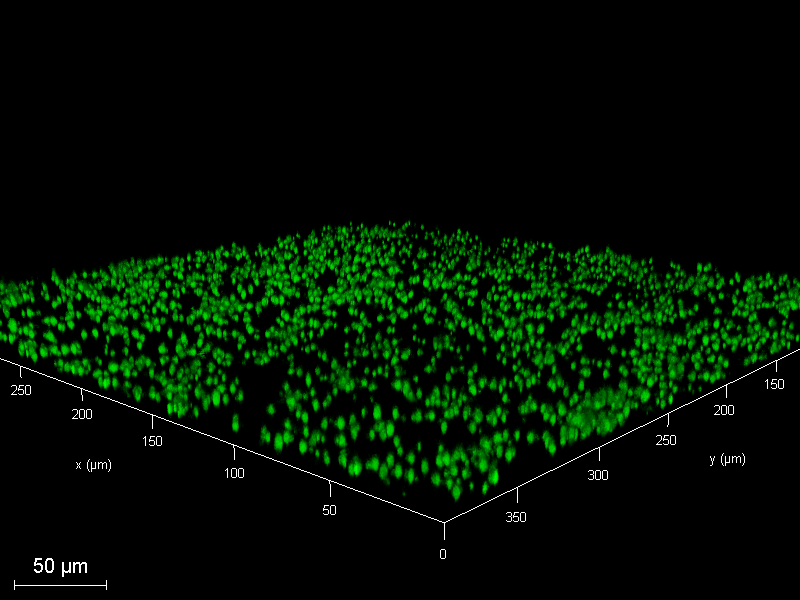

Supplement: Figure 1—source data 1. [file elife-93760-fig1-data1.zip › Figure 1/Figure D Biofilm Image/Fig1D Ca+8uMMgSO4 (3D picture).tif]

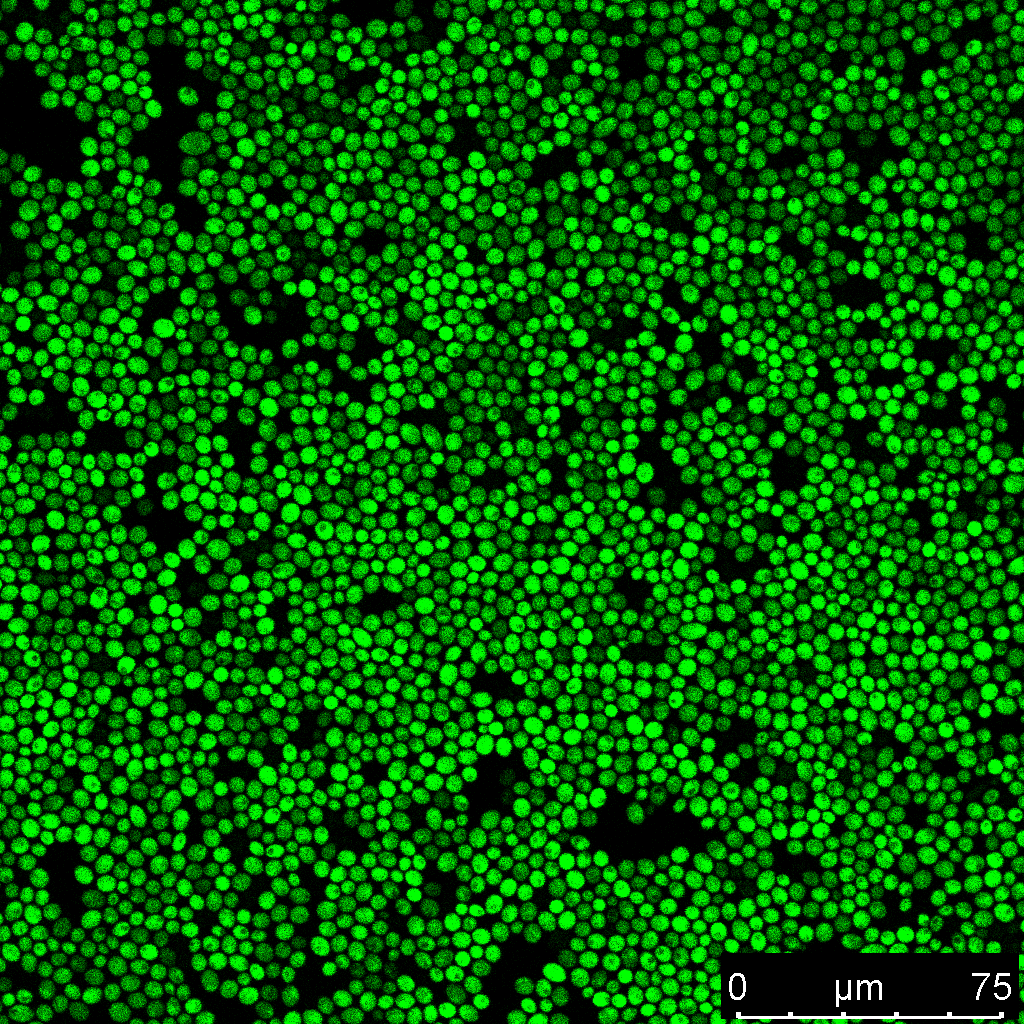

Supplement: Figure 1—source data 1. [file elife-93760-fig1-data1.zip › Figure 1/Figure H Biofilm Image/Fig1H Ca (2D picture).tif]

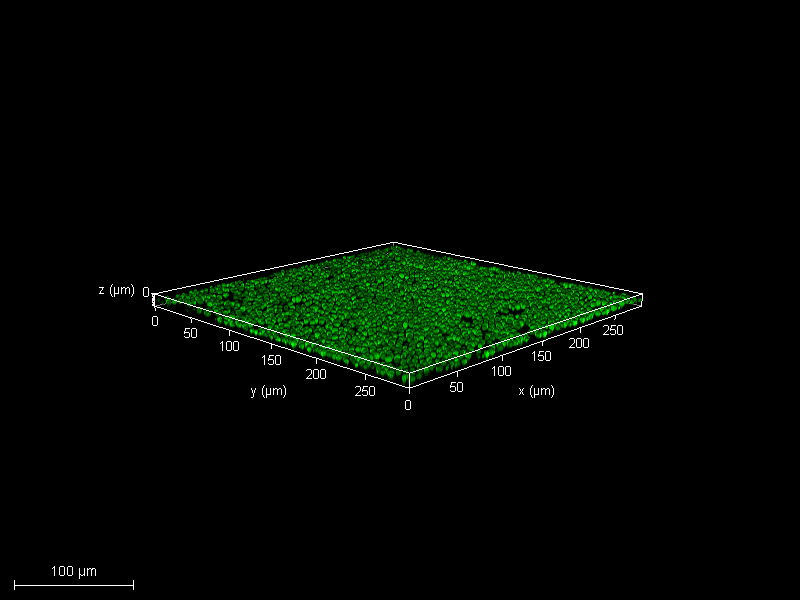

Supplement: Figure 1—source data 1. [file elife-93760-fig1-data1.zip › Figure 1/Figure H Biofilm Image/Fig1H Ca (3D picture).tif]

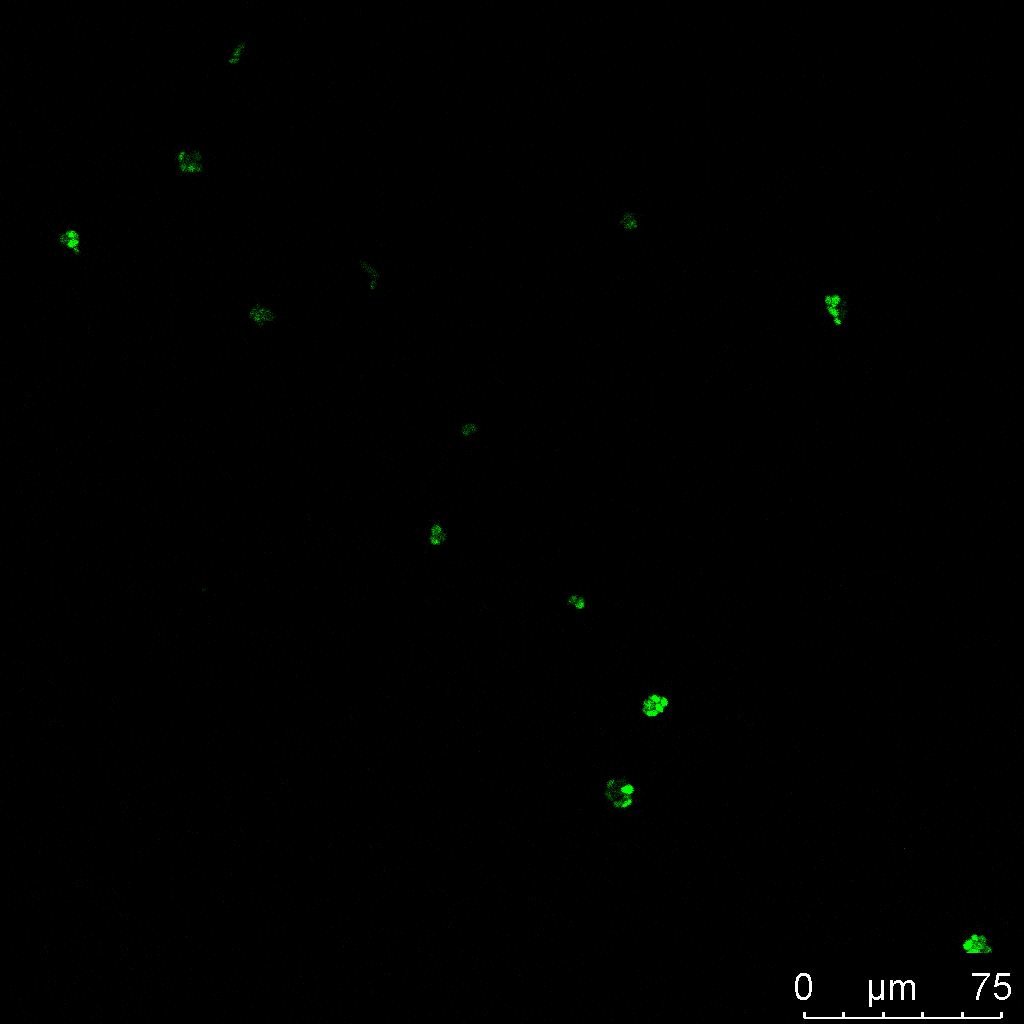

Supplement: Figure 1—source data 1. [file elife-93760-fig1-data1.zip › Figure 1/Figure H Biofilm Image/Fig1H Ca+100uMCE (2D picture).tif]

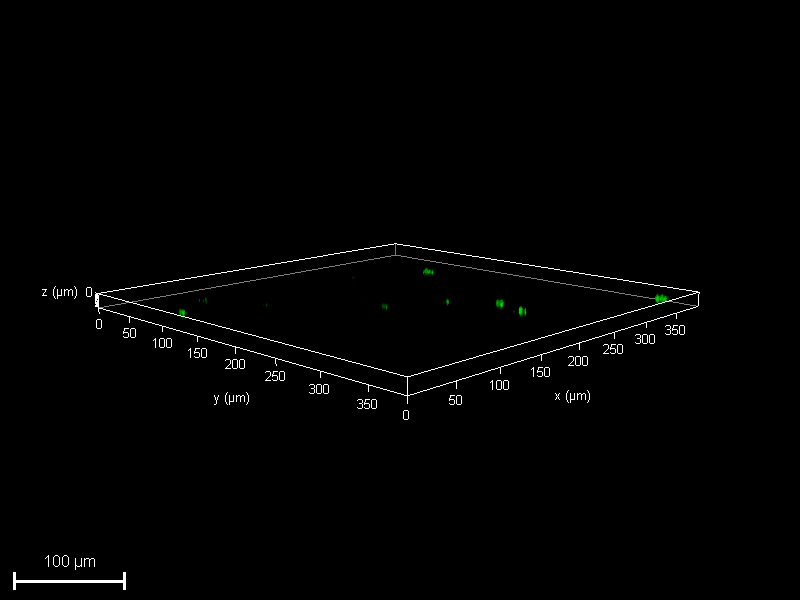

Supplement: Figure 1—source data 1. [file elife-93760-fig1-data1.zip › Figure 1/Figure H Biofilm Image/Fig1H Ca+100uMCE (3D picture).tif]

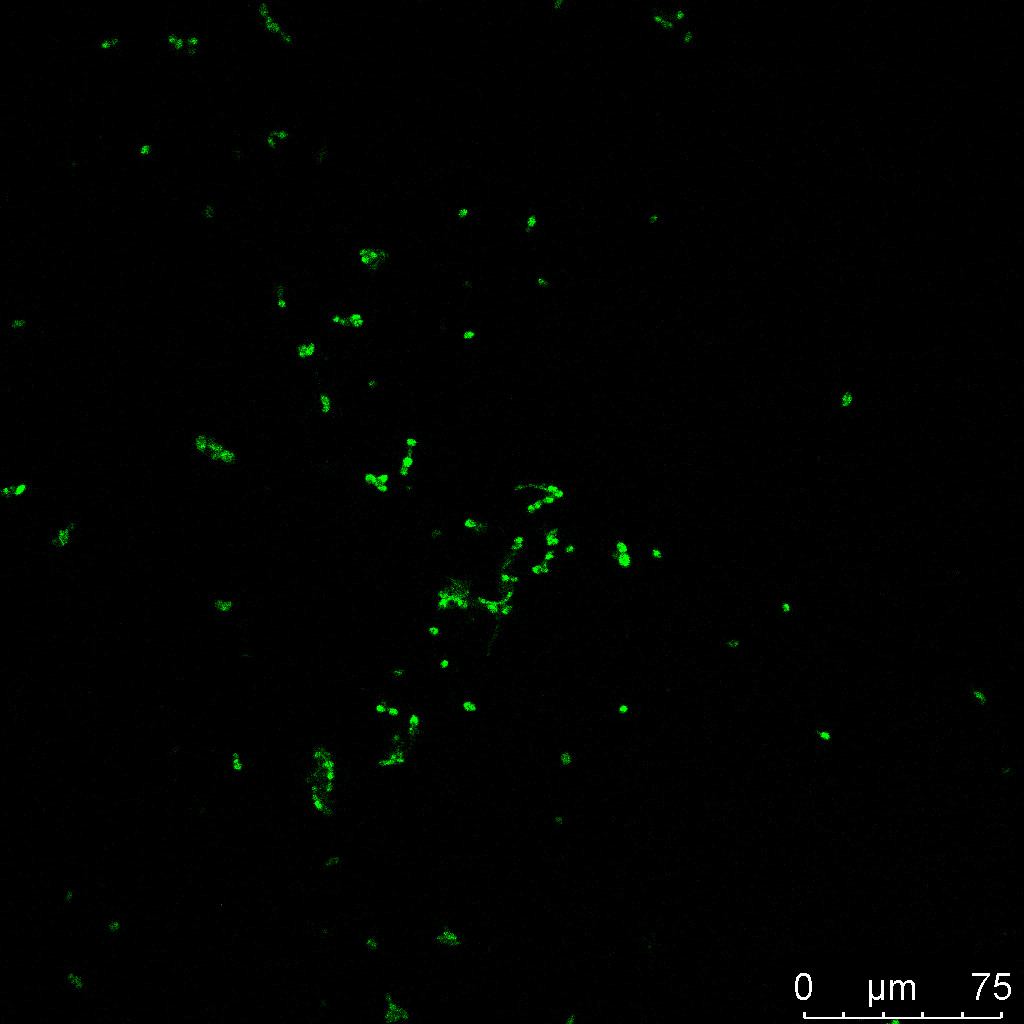

Supplement: Figure 1—source data 1. [file elife-93760-fig1-data1.zip › Figure 1/Figure H Biofilm Image/Fig1H Ca+100uMTPEN (2D picture).tif]

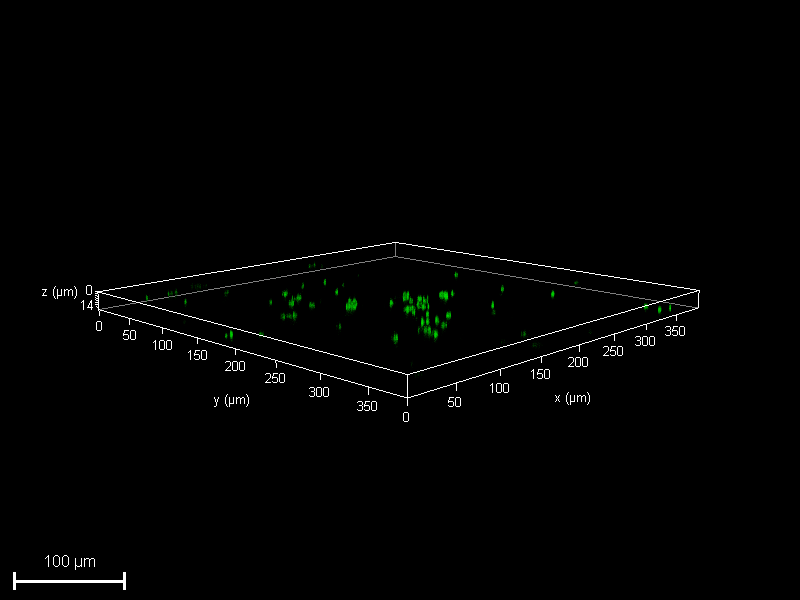

Supplement: Figure 1—source data 1. [file elife-93760-fig1-data1.zip › Figure 1/Figure H Biofilm Image/Fig1H Ca+100uMTPEN (3D picture).tif]

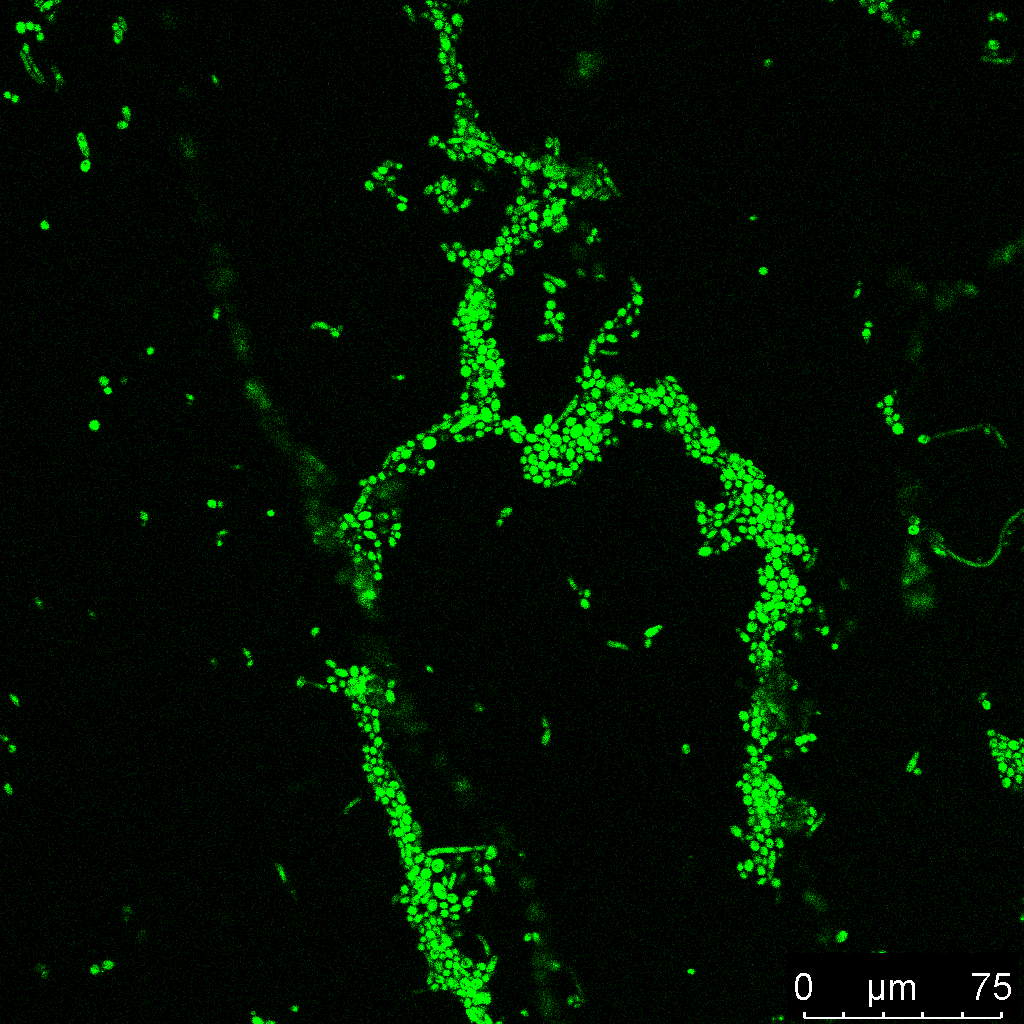

Supplement: Figure 1—source data 1. [file elife-93760-fig1-data1.zip › Figure 1/Figure H Biofilm Image/Fig1H Ca+250uMAprotinin (2D picture).tif]

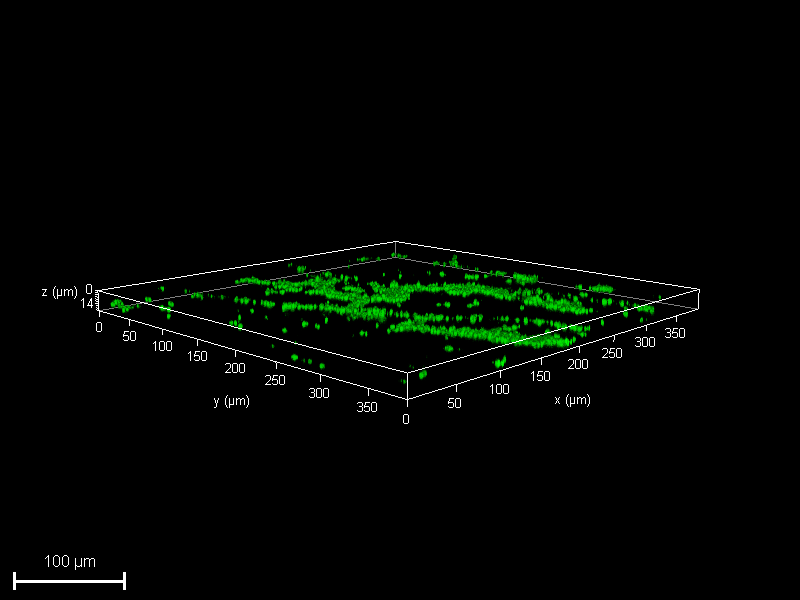

Supplement: Figure 1—source data 1. [file elife-93760-fig1-data1.zip › Figure 1/Figure H Biofilm Image/Fig1H Ca+250uMAprotinin (3D picture).tif]

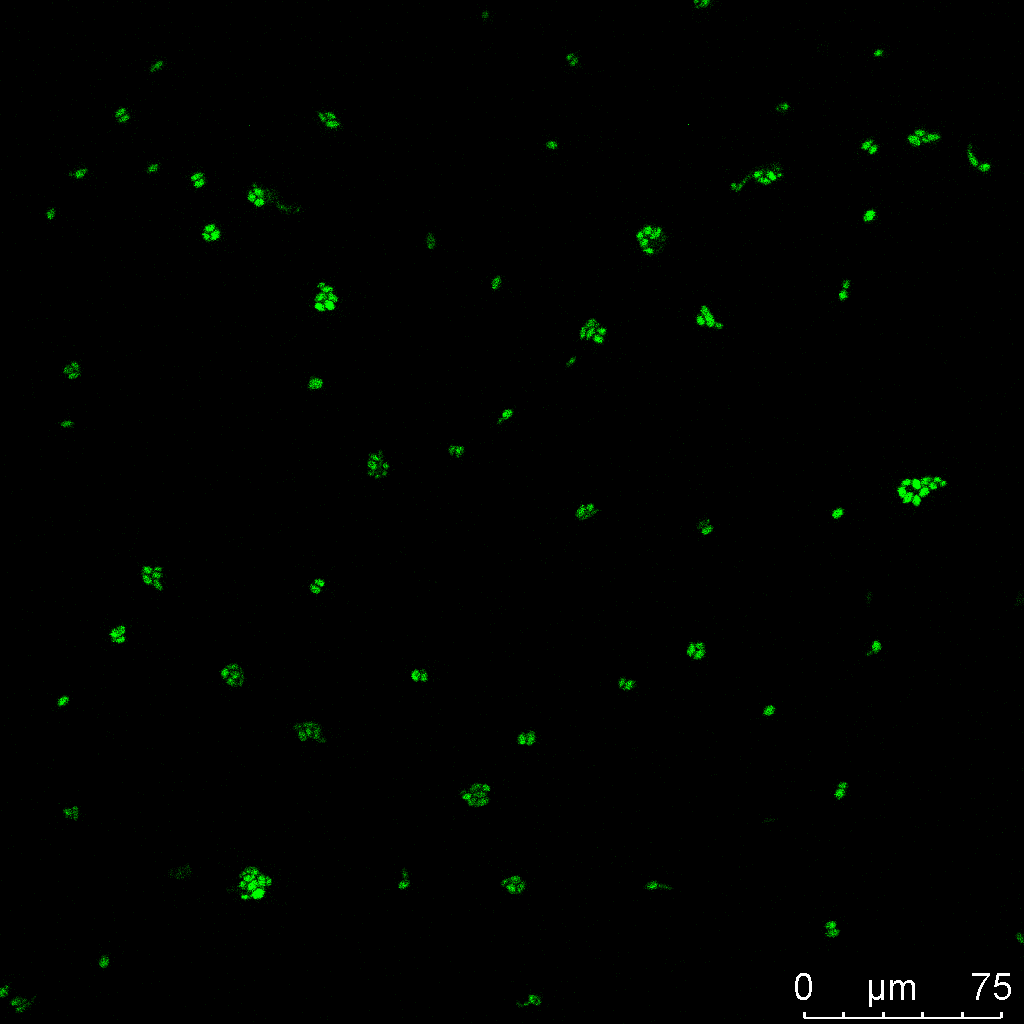

Supplement: Figure 1—source data 1. [file elife-93760-fig1-data1.zip › Figure 1/Figure H Biofilm Image/Fig1H Ca+250uMDTPA (2D picture).tif]

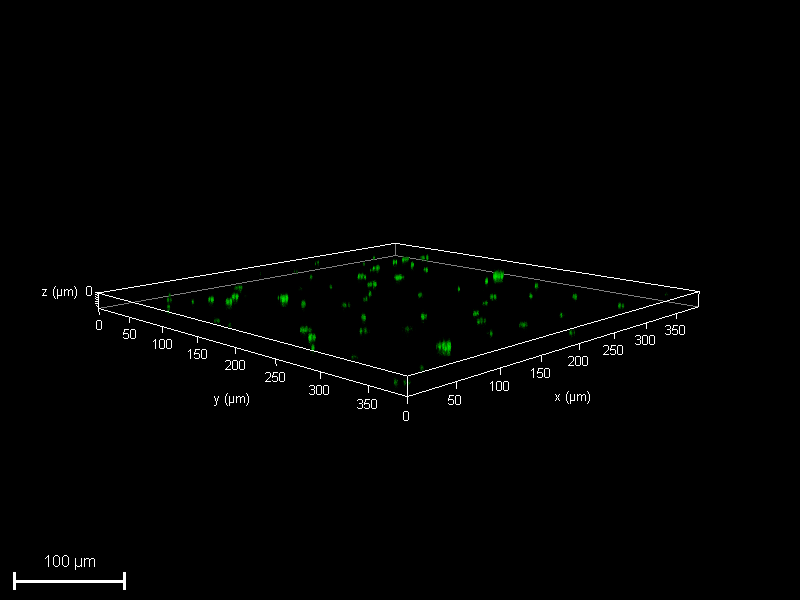

Supplement: Figure 1—source data 1. [file elife-93760-fig1-data1.zip › Figure 1/Figure H Biofilm Image/Fig1H Ca+250uMDTPA (3D picture).tif]

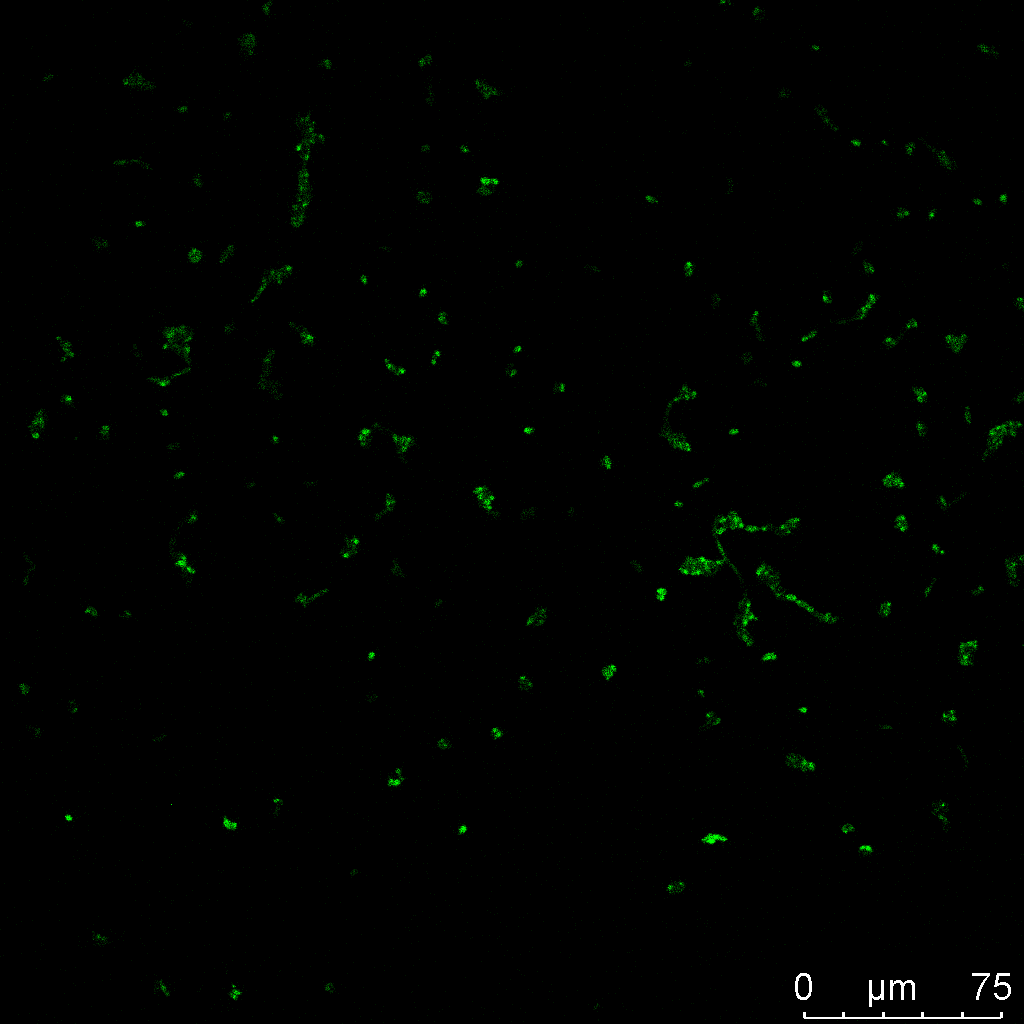

Supplement: Figure 1—source data 1. [file elife-93760-fig1-data1.zip › Figure 1/Figure H Biofilm Image/Fig1H Ca+250uMEDTA (2D picture).tif]

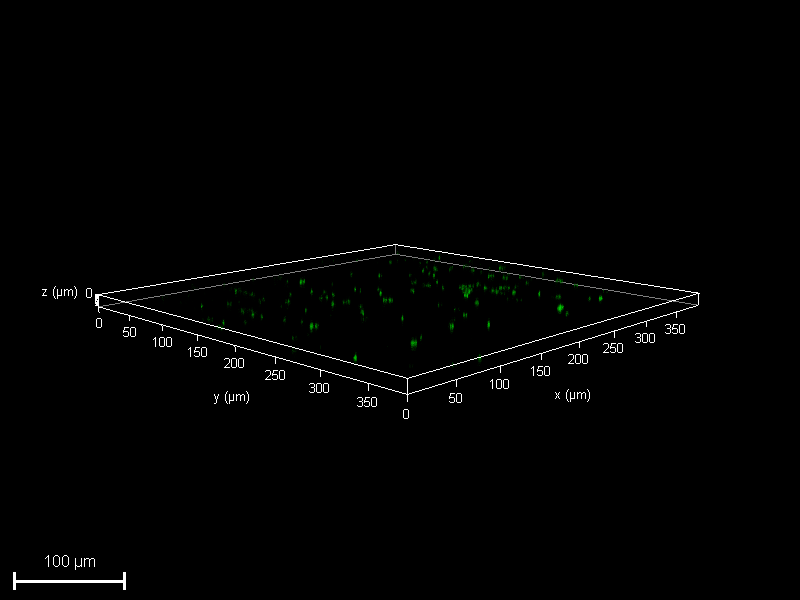

Supplement: Figure 1—source data 1. [file elife-93760-fig1-data1.zip › Figure 1/Figure H Biofilm Image/Fig1H Ca+250uMEDTA (3D picture).tif]

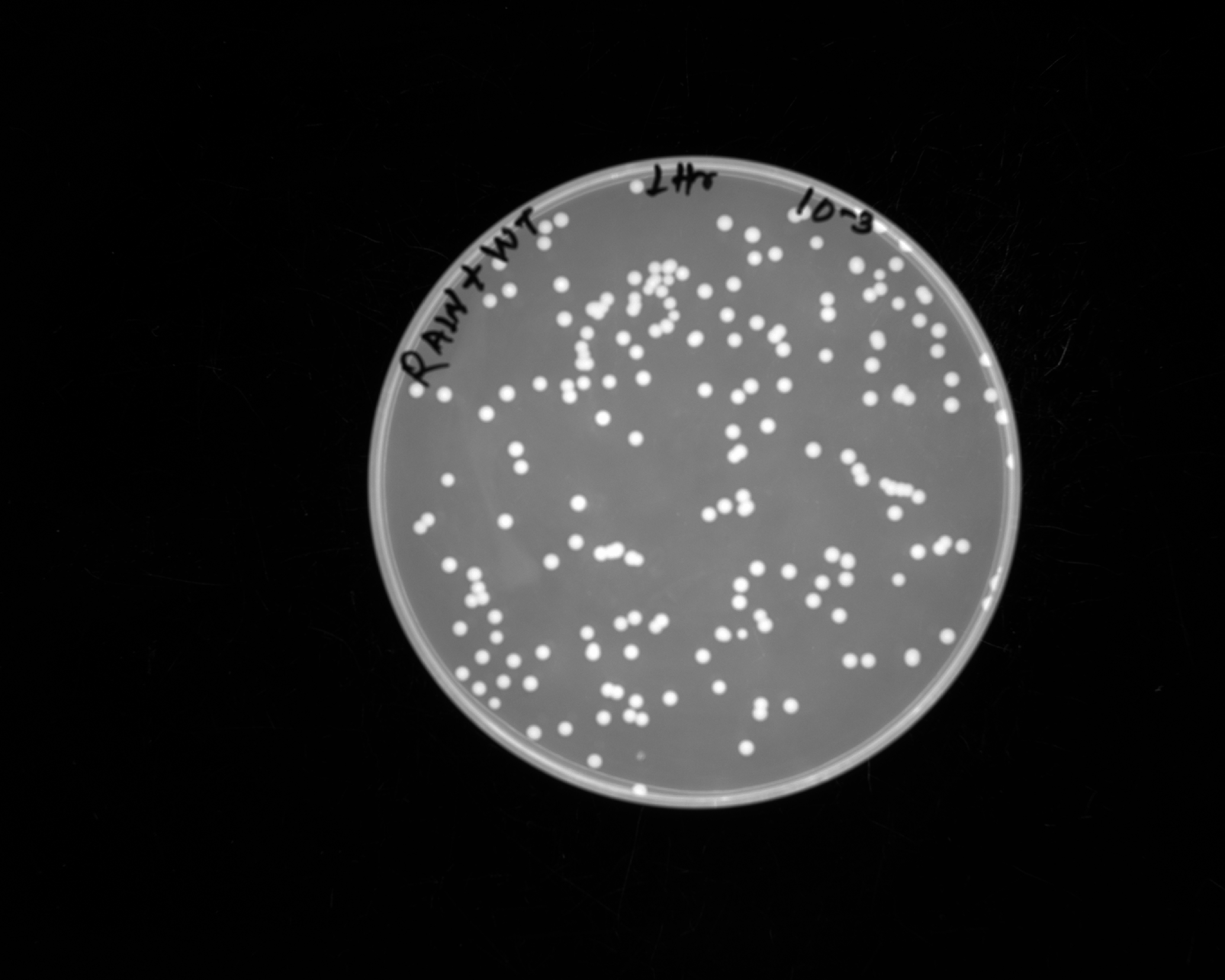

Supplement: Figure 4—source data 1. [file elife-93760-fig4-data1.zip › Figure 4/Fig 4Bi/RAW+Ca 1hr 2023-06-08 10h49m09s(Ethidium Bromide).jpg]

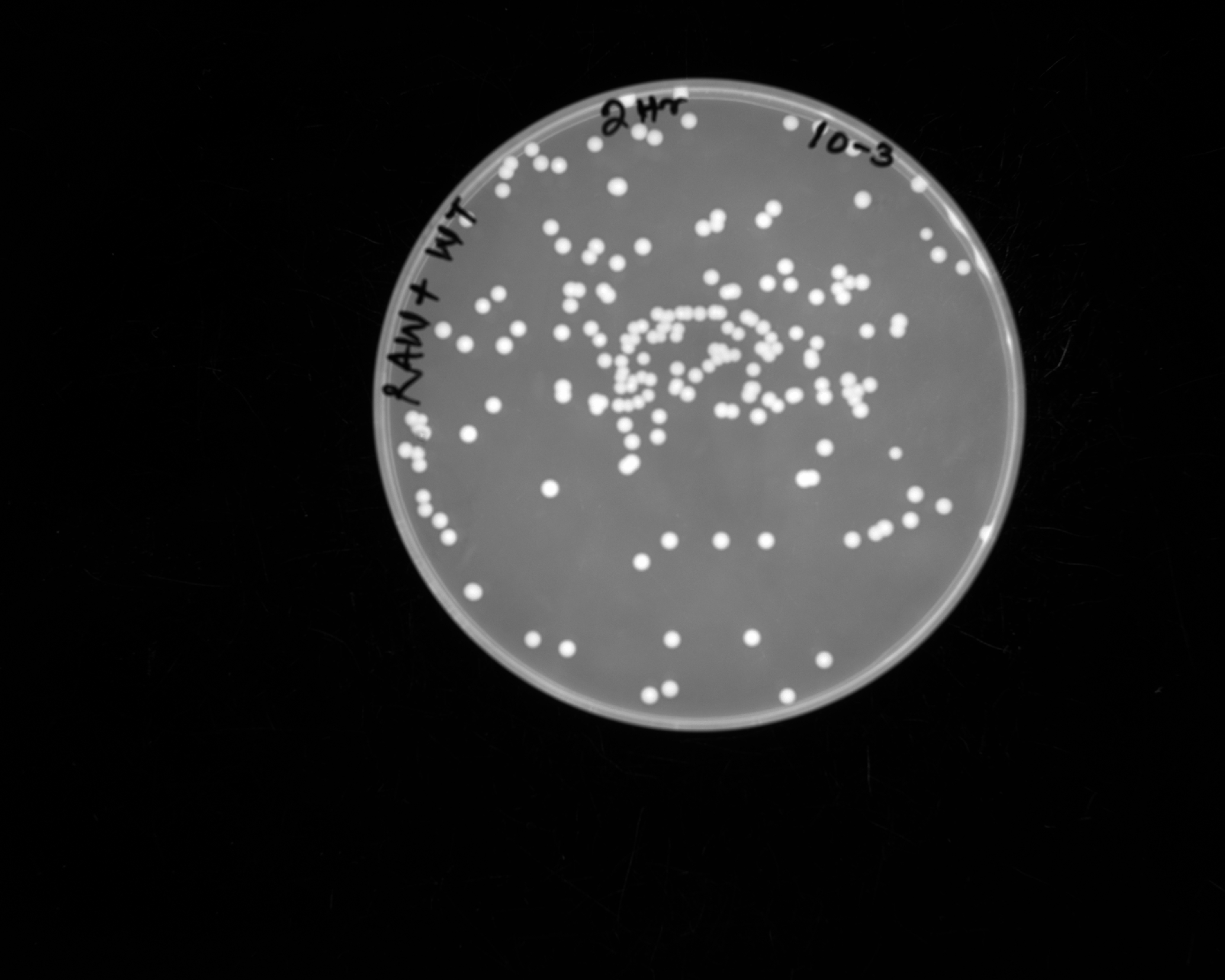

Supplement: Figure 4—source data 1. [file elife-93760-fig4-data1.zip › Figure 4/Fig 4Bi/RAW+Ca 2hr 2023-06-08 10h50m23s(Ethidium Bromide).jpg]

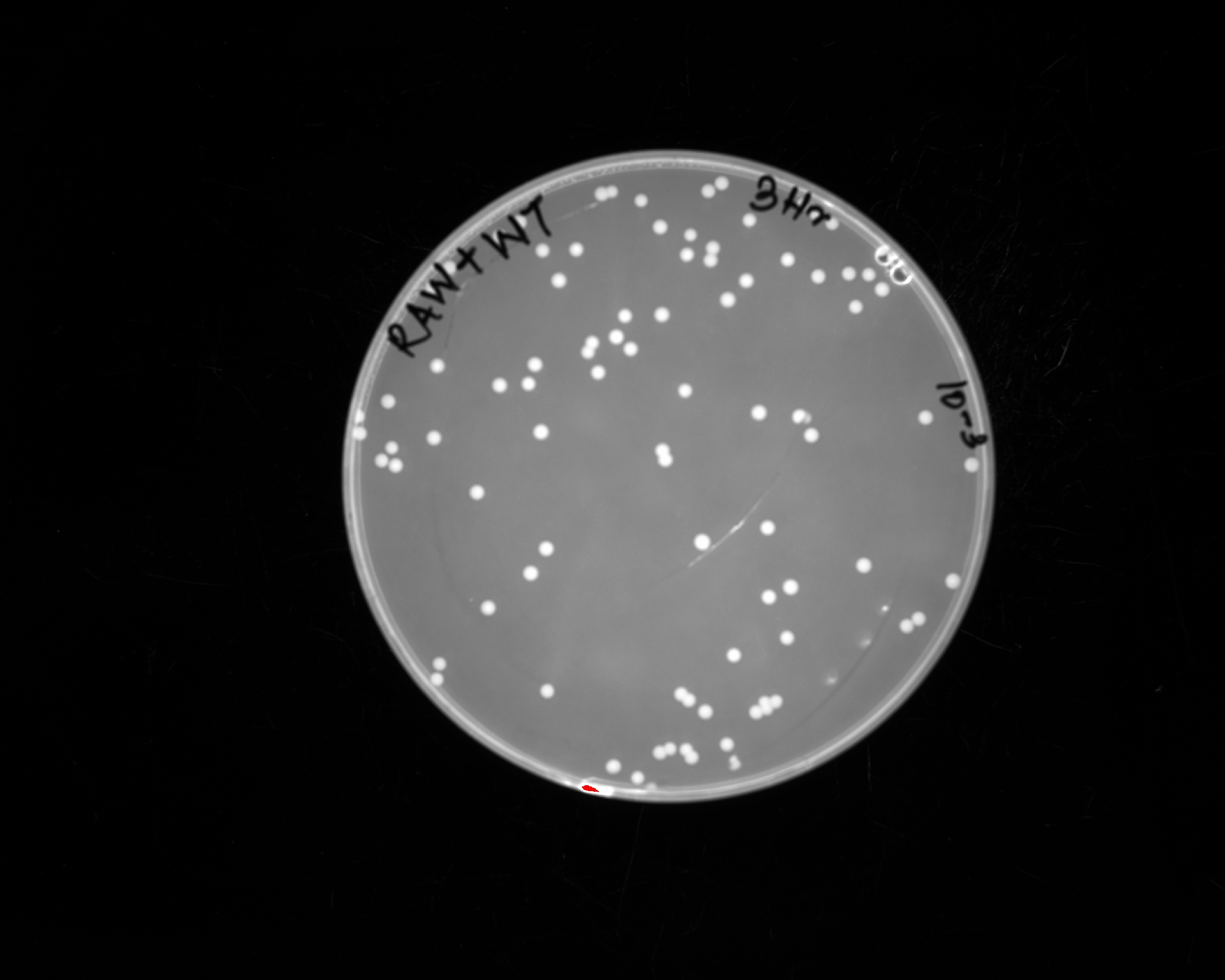

Supplement: Figure 4—source data 1. [file elife-93760-fig4-data1.zip › Figure 4/Fig 4Bi/RAW+Ca 3hr 2023-06-08 10h36m47s(Ethidium Bromide).jpg]

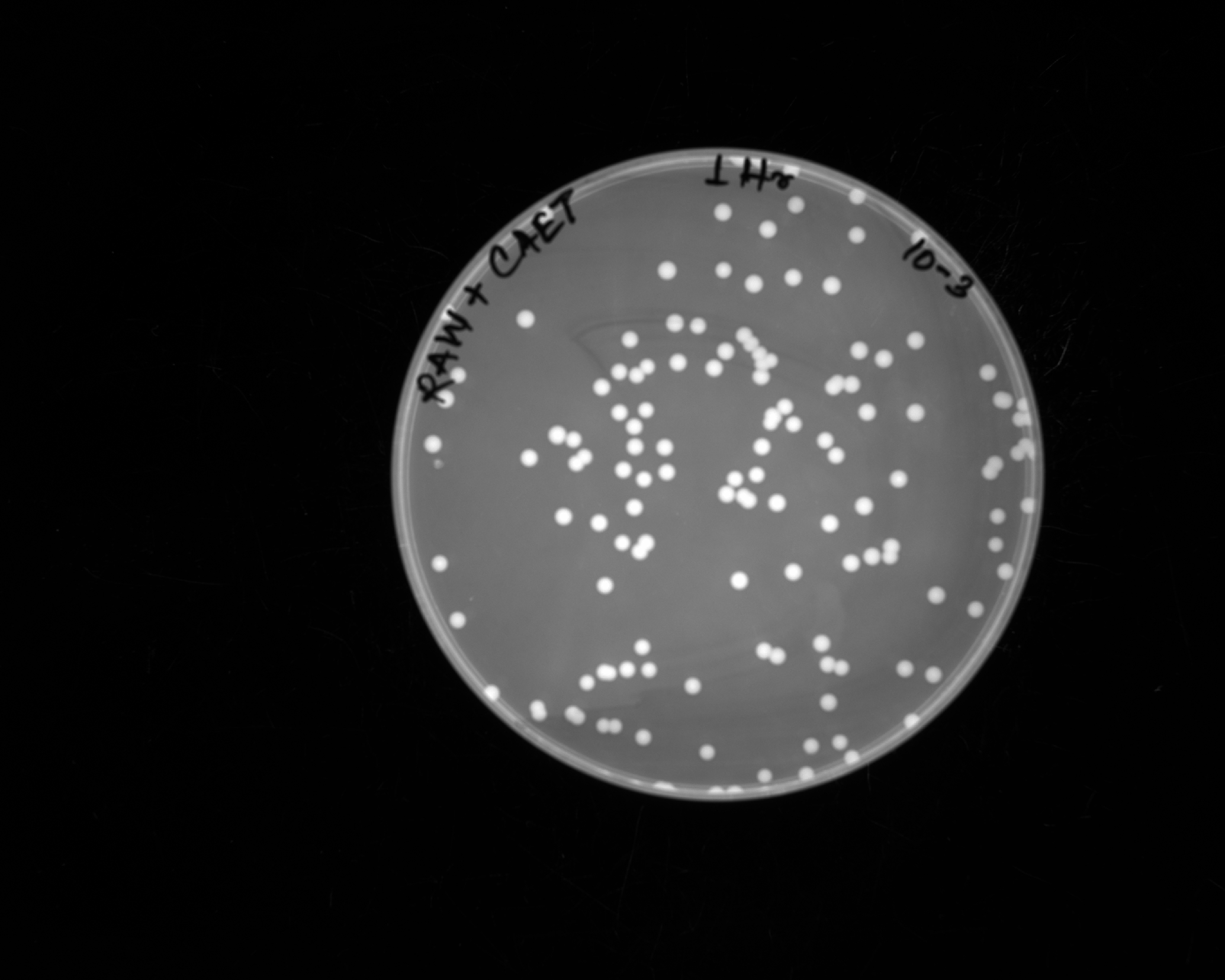

Supplement: Figure 4—source data 1. [file elife-93760-fig4-data1.zip › Figure 4/Fig 4Bi/RAW+CAET 1hr 2023-06-08 10h40m07s(Ethidium Bromide).jpg]

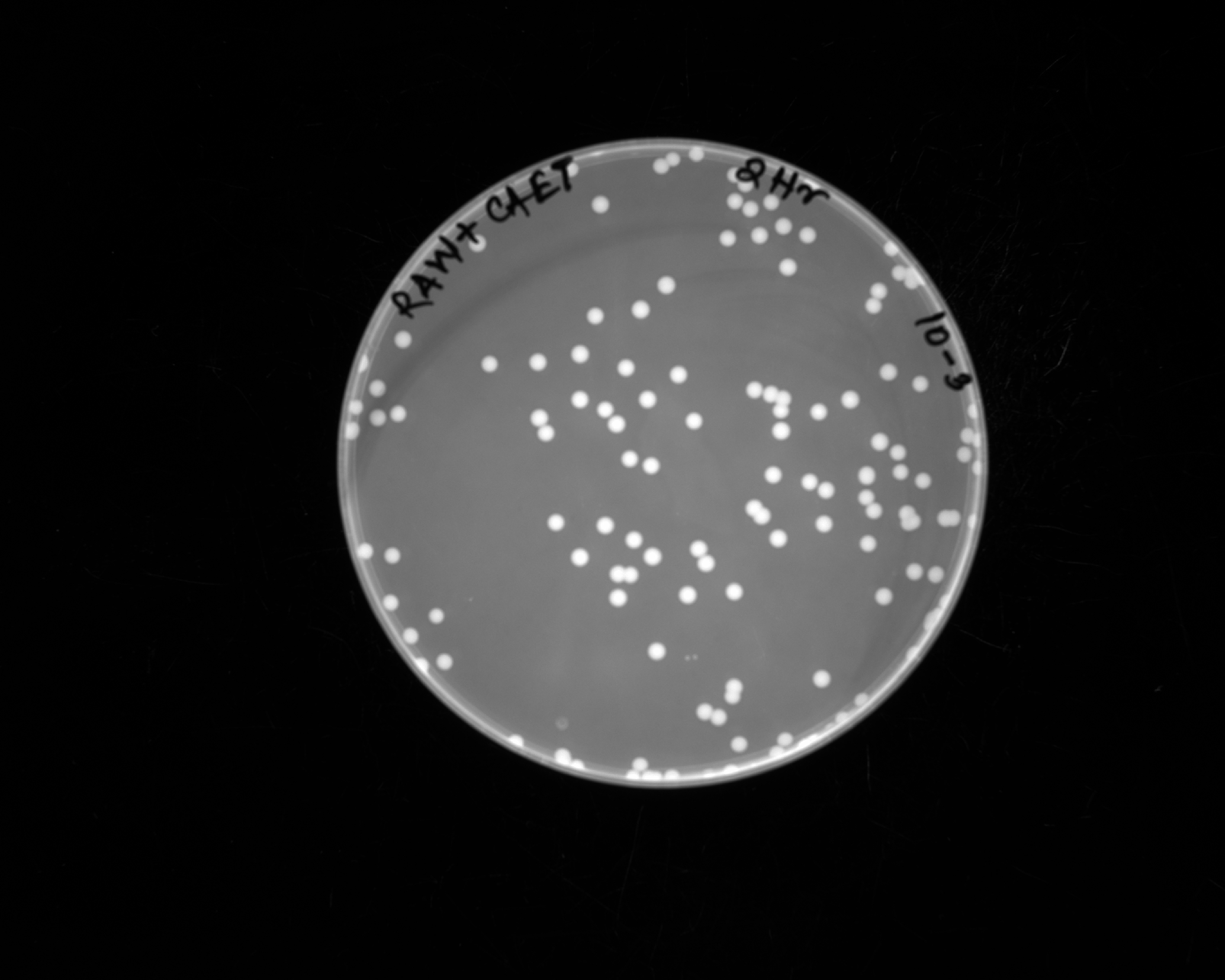

Supplement: Figure 4—source data 1. [file elife-93760-fig4-data1.zip › Figure 4/Fig 4Bi/RAW+CAET 2hr 2023-06-08 10h40m53s(Ethidium Bromide).jpg]

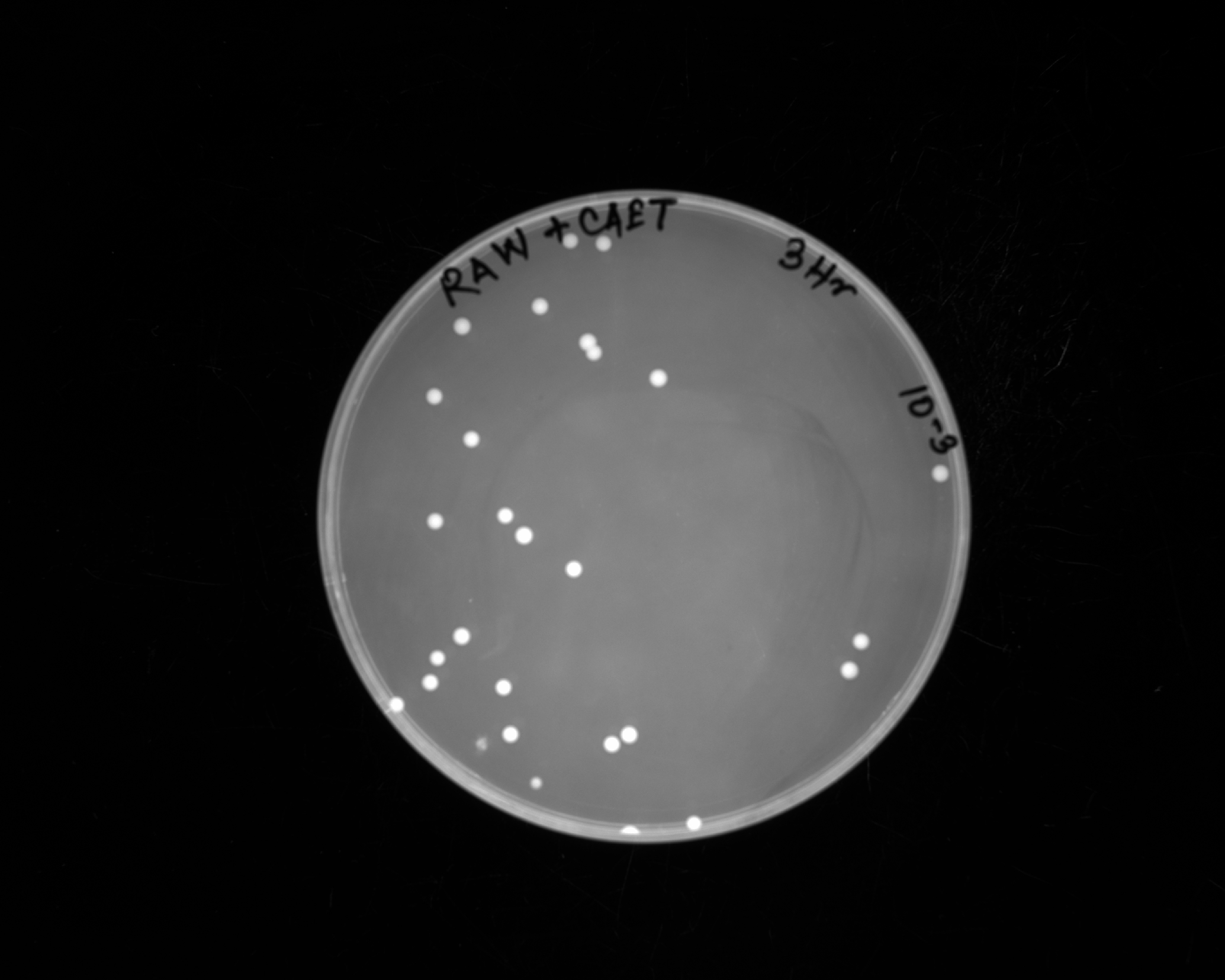

Supplement: Figure 4—source data 1. [file elife-93760-fig4-data1.zip › Figure 4/Fig 4Bi/RAW+CAET 3hr 2023-06-08 10h41m36s(Ethidium Bromide).jpg]

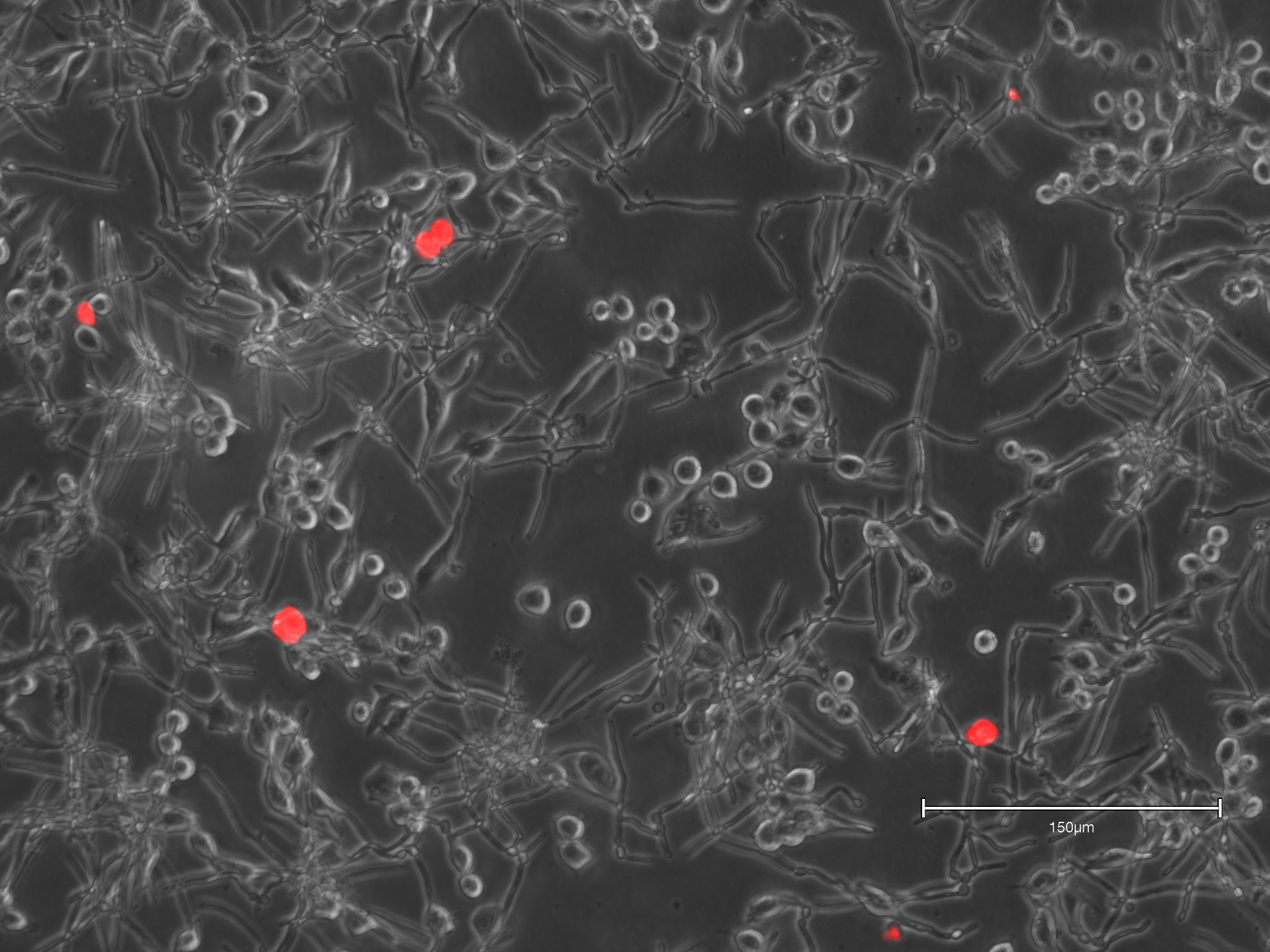

Supplement: Figure 4—source data 1. [file elife-93760-fig4-data1.zip › Figure 4/Fig 4Ci/CAET 20X.jpg]

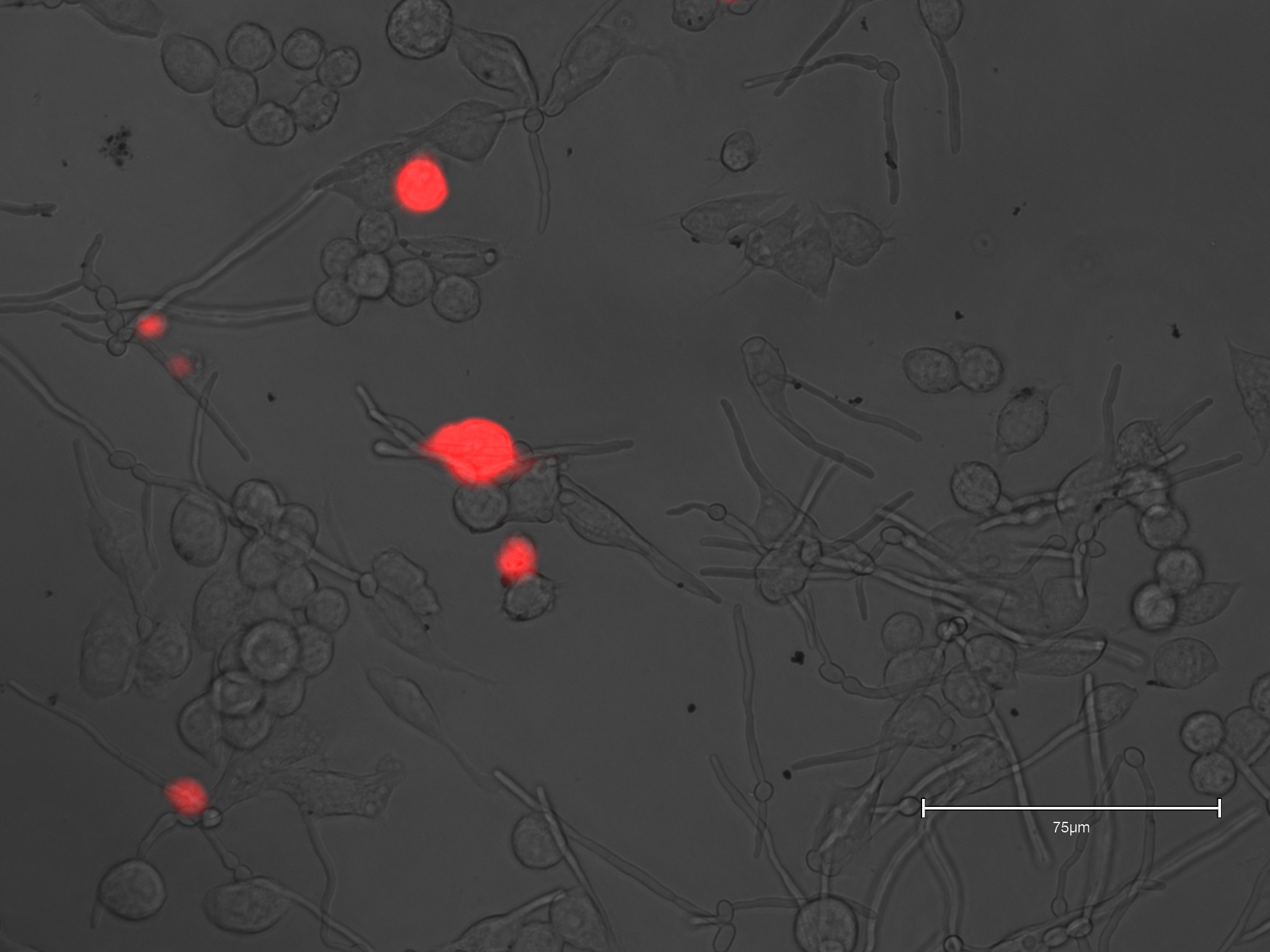

Supplement: Figure 4—source data 1. [file elife-93760-fig4-data1.zip › Figure 4/Fig 4Ci/CAET 40X.jpg]

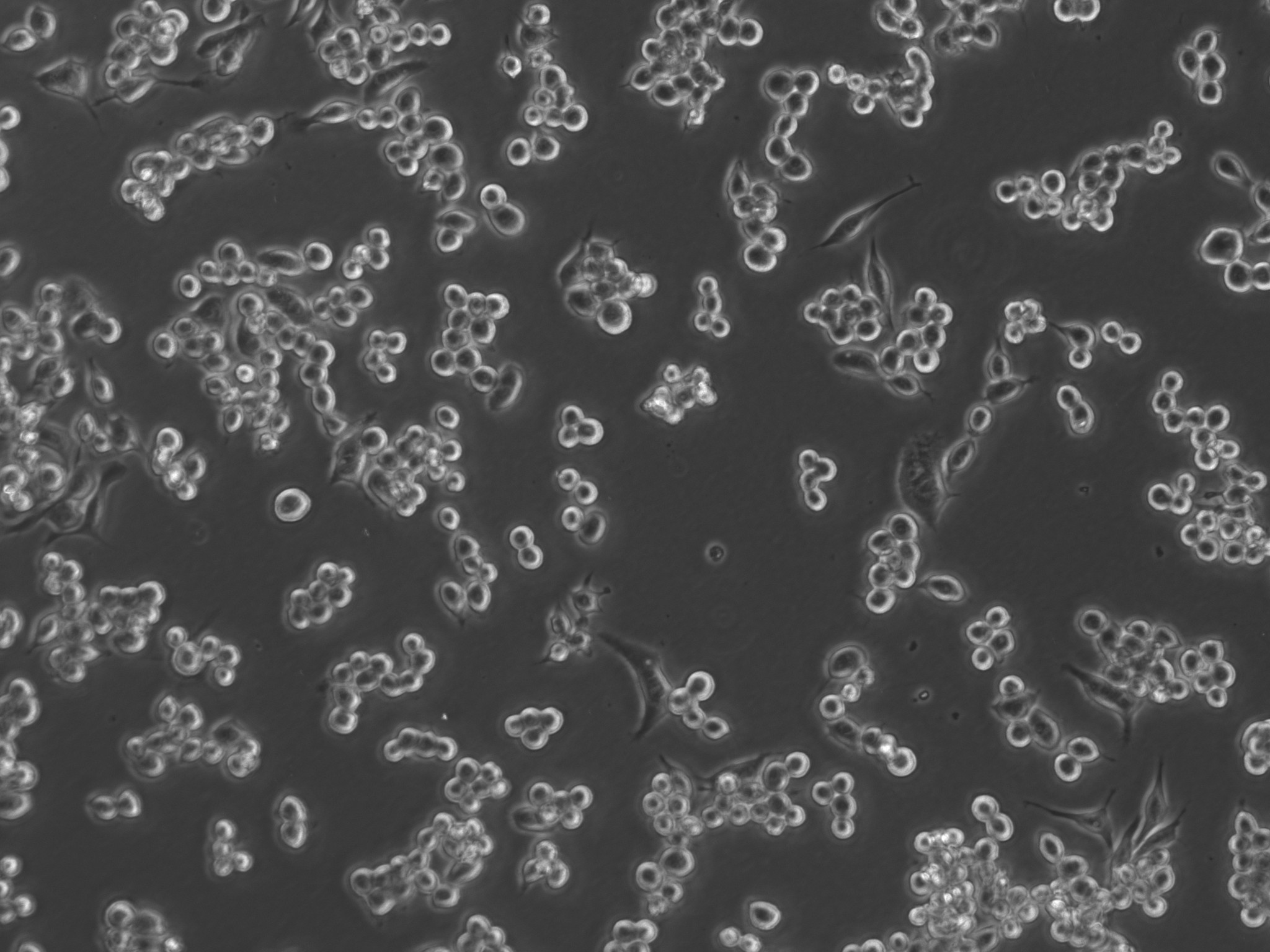

Supplement: Figure 4—source data 1. [file elife-93760-fig4-data1.zip › Figure 4/Fig 4Ci/RAW 20X.jpg]

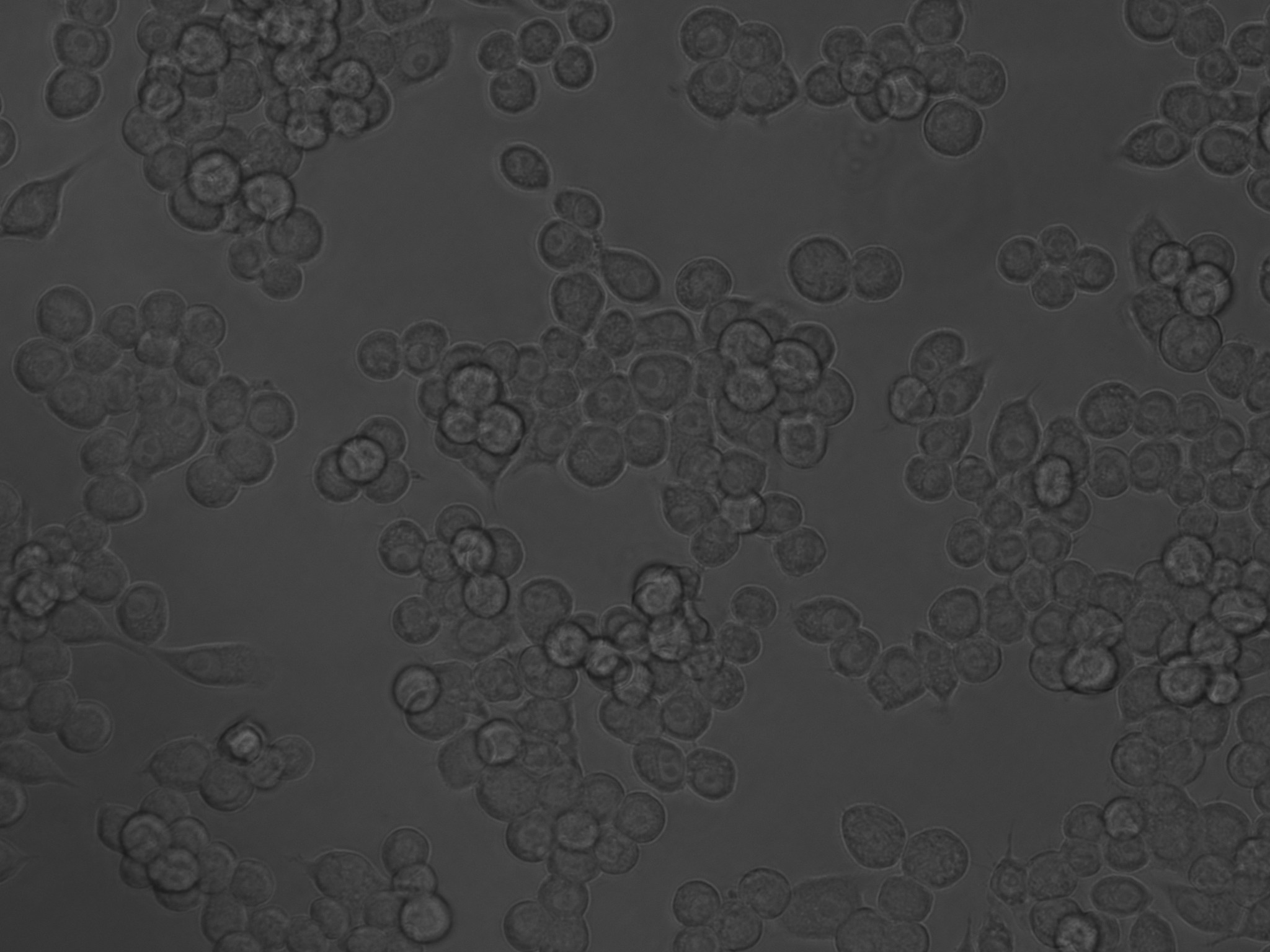

Supplement: Figure 4—source data 1. [file elife-93760-fig4-data1.zip › Figure 4/Fig 4Ci/RAW 40X.jpg]

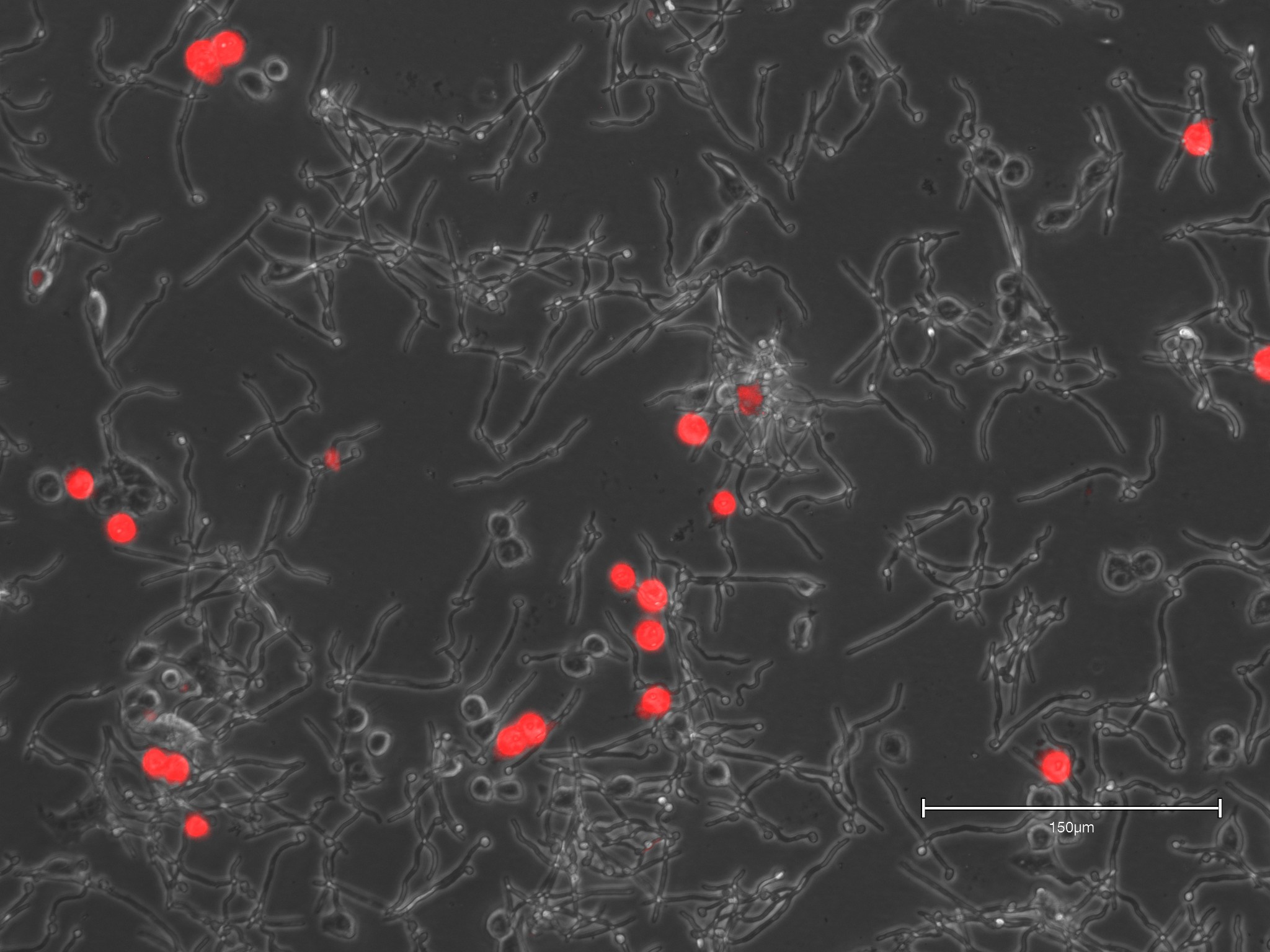

Supplement: Figure 4—source data 1. [file elife-93760-fig4-data1.zip › Figure 4/Fig 4Ci/WT 20X.jpg]

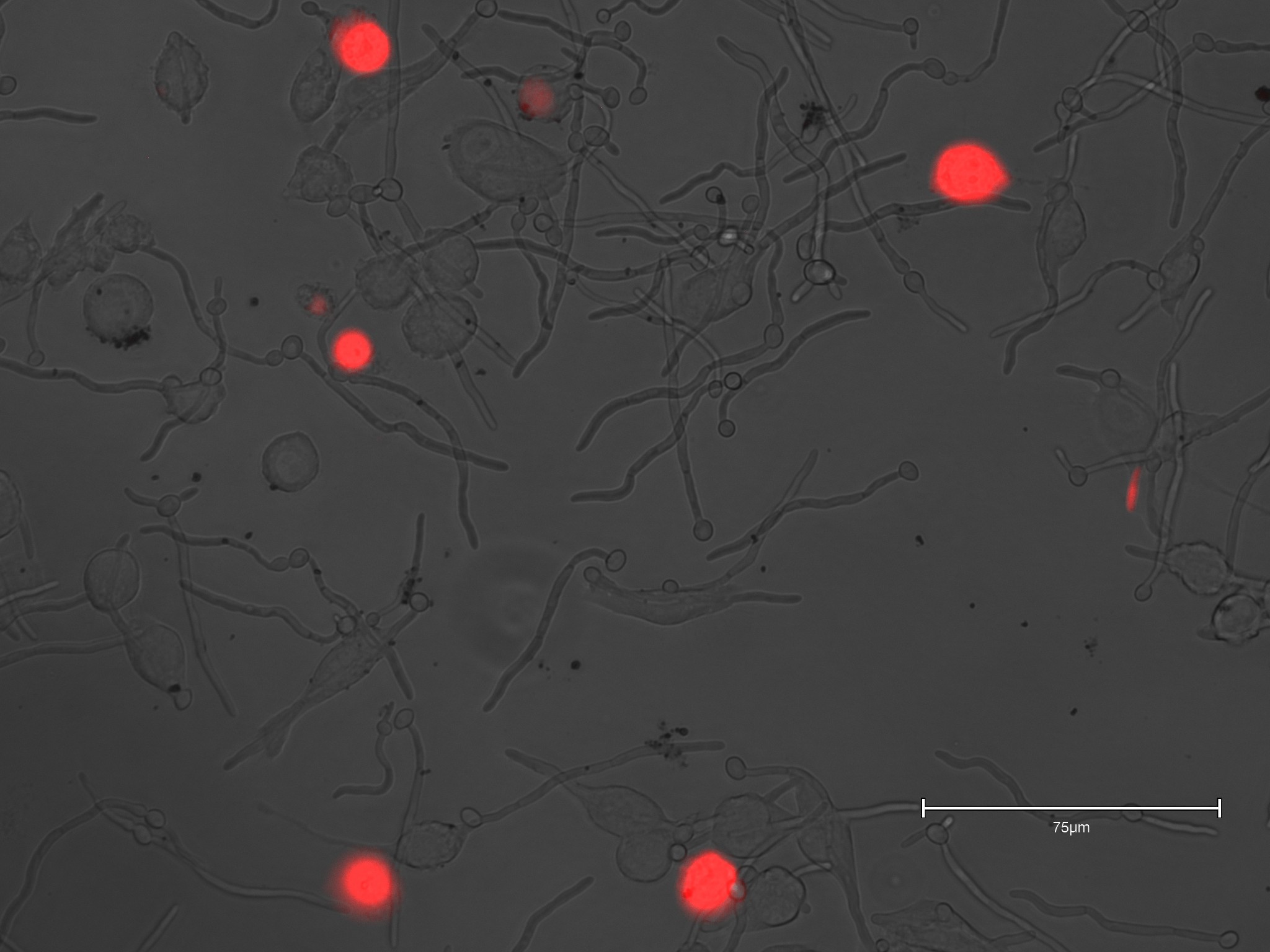

Supplement: Figure 4—source data 1. [file elife-93760-fig4-data1.zip › Figure 4/Fig 4Ci/WT 40X.jpg]

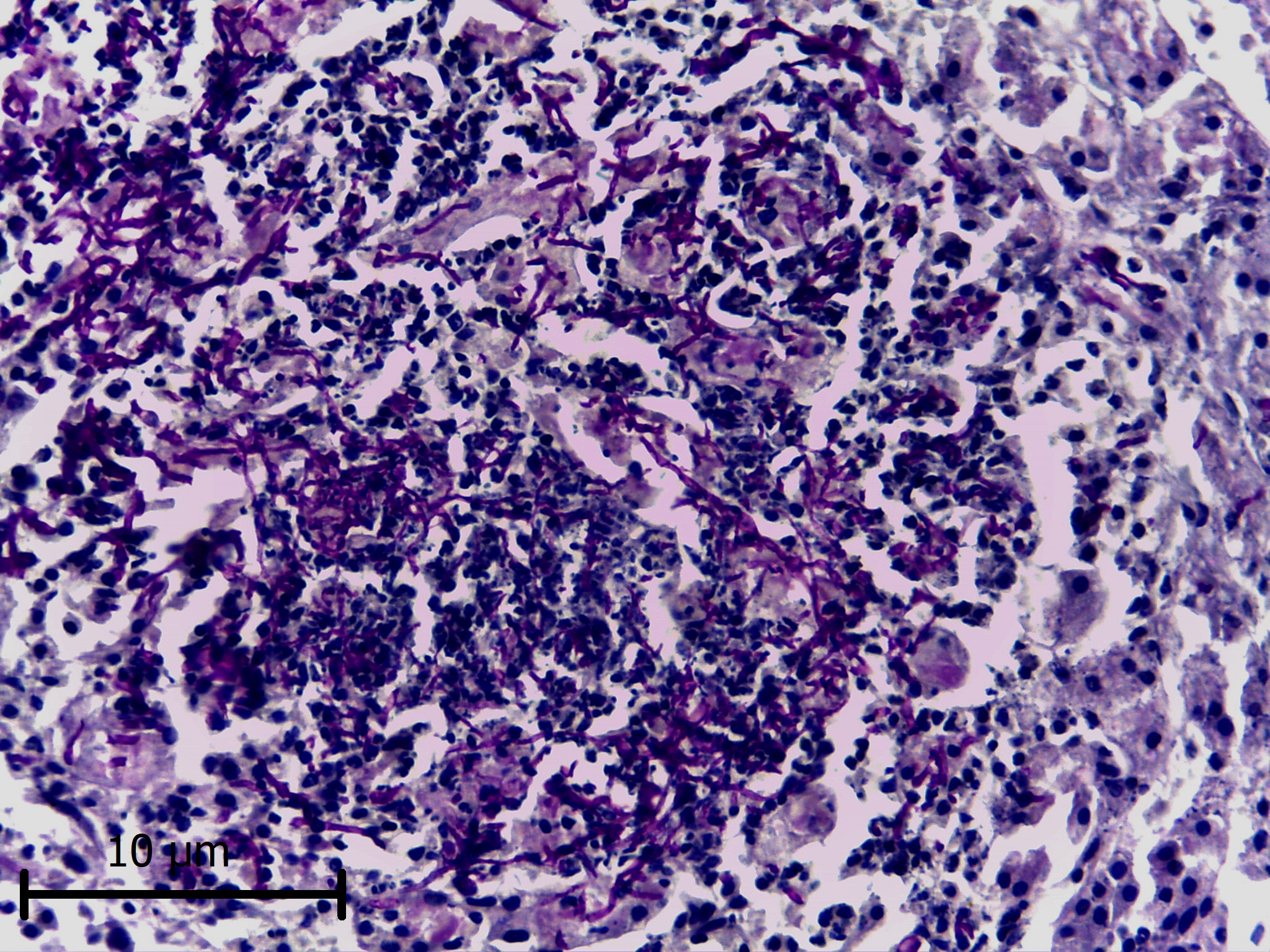

Supplement: Figure 5—source data 1. [file elife-93760-fig5-data1.zip › Figure 5/Figure 5 C/1° Saline-2° Ca Medulla 40X.jpg]

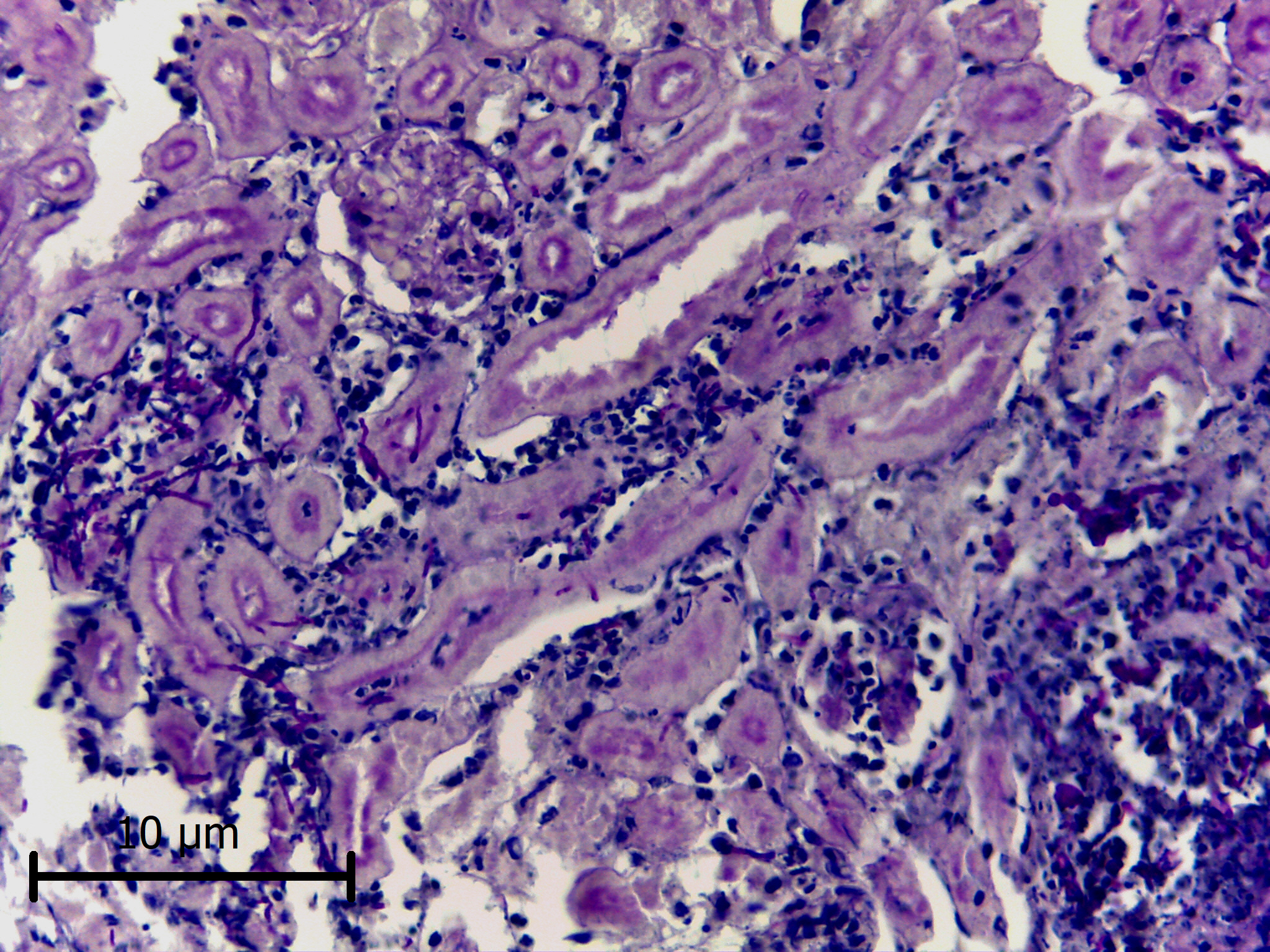

Supplement: Figure 5—source data 1. [file elife-93760-fig5-data1.zip › Figure 5/Figure 5 C/1° Saline-2° Ca Cortex 40X.jpg]

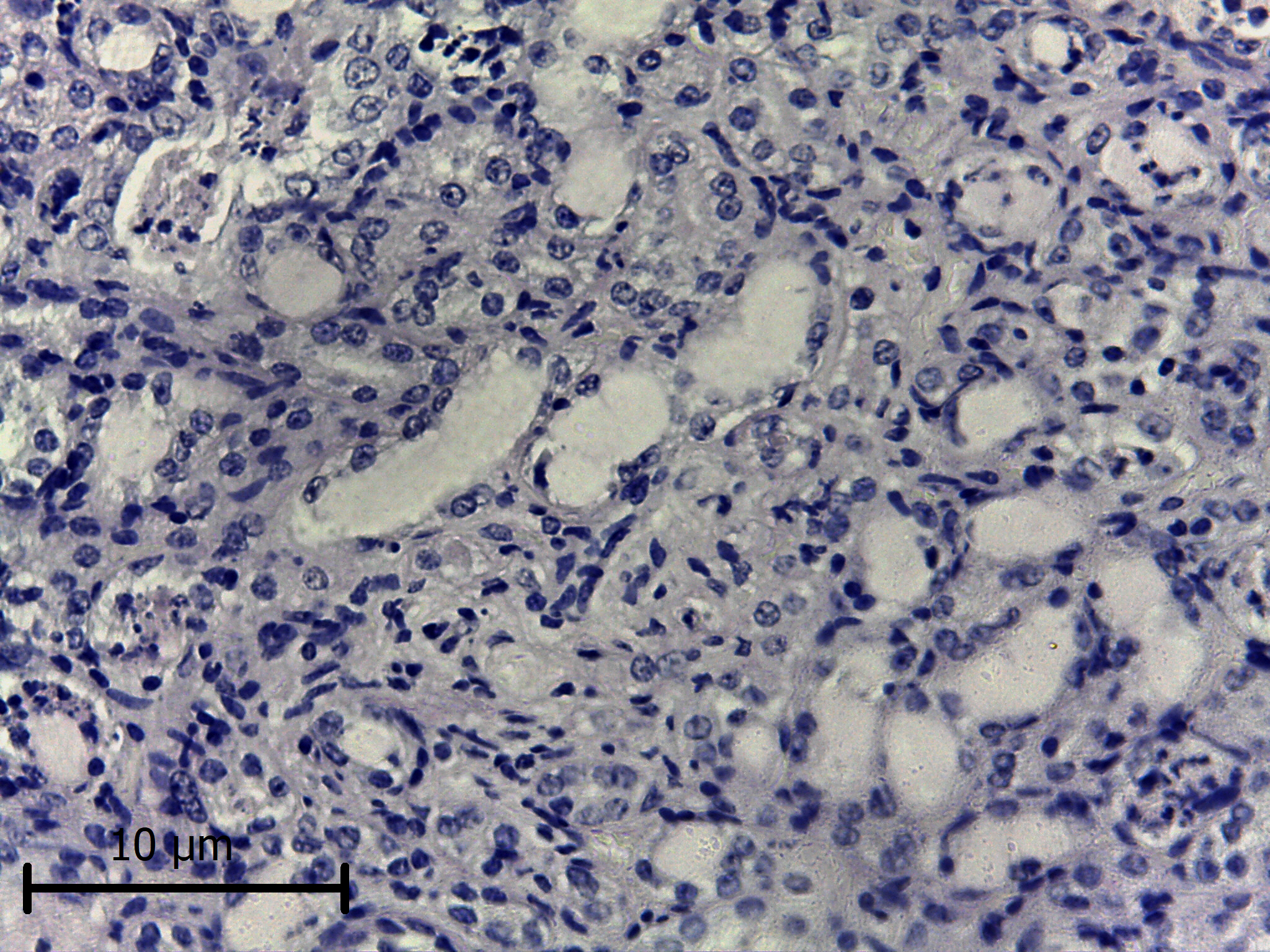

Supplement: Figure 5—source data 1. [file elife-93760-fig5-data1.zip › Figure 5/Figure 5 C/Ca Cortex 40X.jpg]

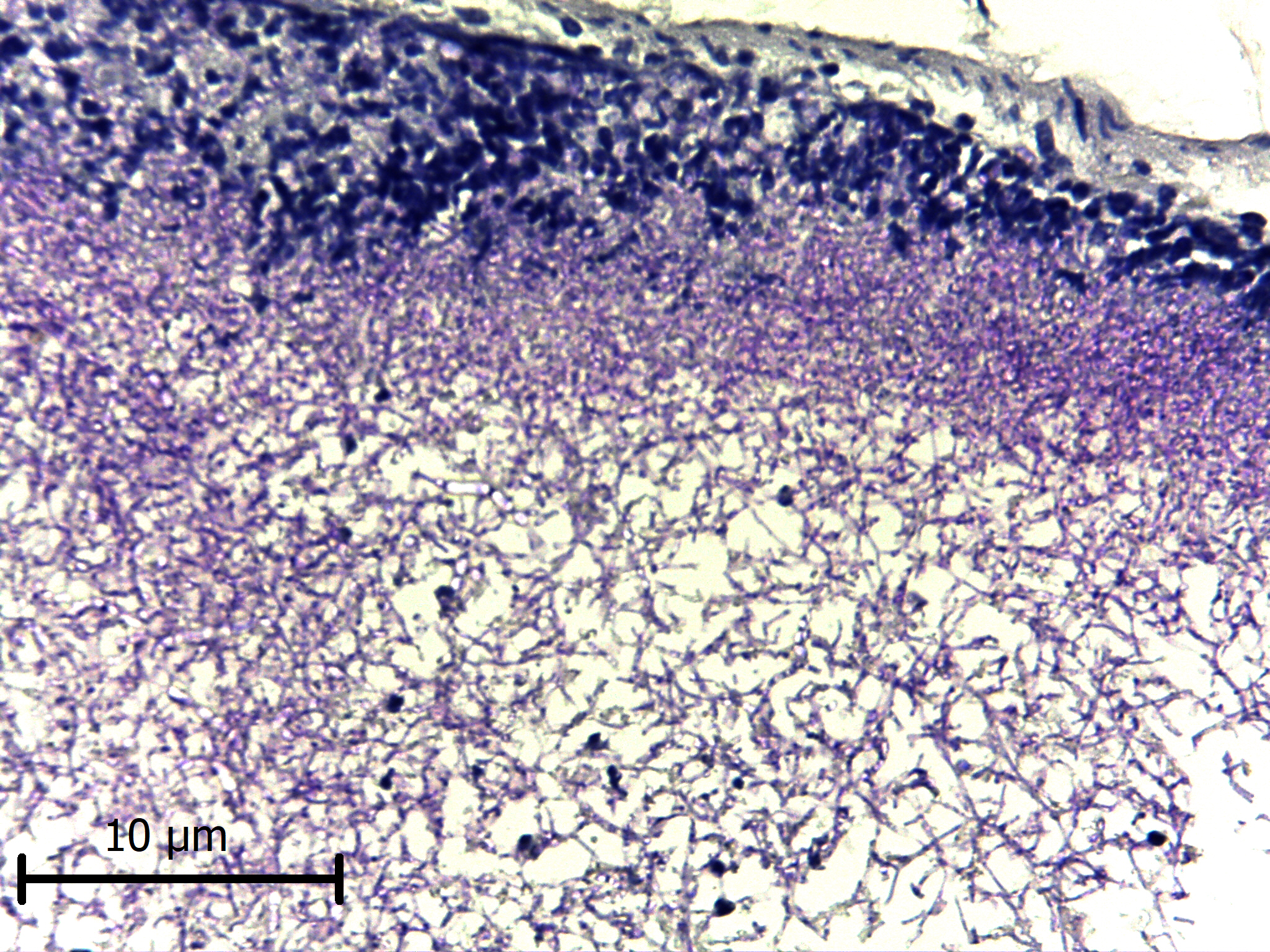

Supplement: Figure 5—source data 1. [file elife-93760-fig5-data1.zip › Figure 5/Figure 5 C/Ca Medulla 40X.jpg]
